# Constructing Activatable Photosensitizers Using Covalently Modified Mesoporous Silica

**DOI:** 10.1002/advs.202406887

**Published:** 2025-01-15

**Authors:** Yan Liu, Xiang Wang, Ben Wang, Zhenni Lu, Changru Wu, Zhanghao He, Libo Jiang, Peng Wei, Tao Yi

**Affiliations:** ^1^ State Key Laboratory for Modification of Chemical Fibers and Polymer Materials College of Chemistry and Chemical Engineering Donghua University Shanghai 201620 China; ^2^ Department of Orthopaedic Surgery Zhongshan Hospital Fudan University Shanghai 200032 China; ^3^ Present address: Orthopedic Department Taizhou Hospital of Zhejiang Province Taizhou Zhejiang 317000 China

**Keywords:** activatable photosensitizer, combination therapy, mesoporous silica, photodynamic therapy

## Abstract

The combination of photosensitizers (PSs) and nanomaterials is a widely used strategy to enhance PS efficacy and broaden their applicability. However, the current nanocarrier‐based delivery strategies focus on conventional PSs, neglecting the critical issue of PS phototoxicity. In this study, DHUOCl‐25, an activatable PS (aPS) activated by hypochlorous acid, is synthesized by combining a silicon source structure and an activation unit. DHUOCl‐25 functions as a silica source for synthesizing activatable mesoporous silica nanostrctures (MSNs) using a standardized protocol, enabling the synthesis of aPS‐covalently modified MSNs for a variety of biologic therapeutic applications. On one hand, the resulting nano‐aPS maintains aPS functionality for antibacterial application by achieving synergistic antibacterial action via MB PDT and retained cetyltrimethylammonium bromide (CTAB) (antibacterial agent) (DHU–MSNs‐2). On the other hand, the nano‐aPS exhibits MSN properties for drug loading, facilitating the synergistic integration of photodynamic therapy with chemotherapy (DHU–MSNs‐6) of tumors and demonstrating efficacy against the spinal metastases of lung cancer. These results validate this strategy for developing novel aPSs and expanding the application of aPSs and MSNs.

## Graphical abstract

This study presents an activatable photosensitizer (aPS, DHUOCl‐25) activated by hypochlorous acid, which acts as a silica source for the fabrication of mesoporous silica nanostructures (MSNs). The resulting nano‐aPS exhibits antibacterial properties and effectively integrates photodynamic therapy and chemotherapy for the treatment of cancer, meeting diverse therapeutic requirements and highlighting the potential of aPSs and MSNs in clinical applications.

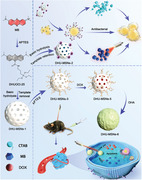

## 1 Introduction

Photodynamic therapy (PDT) employs “photosensitizers (PSs)” and “light” to treat diseases.^[^ ^1^ ^]^ Under specific light wavelengths, PSs are activated to generate highly reactive oxygen species (ROS), achieving therapeutic effects.^[^ ^2^ ^]^ PDT is advantageous over chemotherapy and surgical and radiation therapies owing to its simplicity, noninvasiveness, lack of drug resistance, safety, and minimal adverse effects. Therefore, it is a prominent alternative for antibacterial and anticancer applications.^[^ ^3^ ^]^ However, conventional PSs often lack tissue or cell specificity, causing phototoxicity and limiting PDT application.^[^ ^4^ ^]^ To address this issue, studies have explored targeted PS delivery via nanoplatforms, including liposomes and polymer micelles.^[^ ^5^ ^]^ Nano delivery systems based on mesoporous silica nanostructures (MSNs) leverage their high specific surface area, tunable pore size, and customizable morphology to enhance mechanical and thermal stability, as well as biocompatibility.^[^ ^6^ ^]^ Also, MSNs were used in anti‐biofilm with some results.^[^ ^7^ ^]^ Integrating MSNs with PSs has yielded therapeutic constructs that enhance water solubility, promote PS accumulation at pathological sites, and enable the codelivery of drugs, thereby augmenting therapeutic potency.^[^ ^8^ ^]^ However, whether the PS is covalently anchored or physically embedded, the reported MSN carriers cannot completely suppress the photosensitizing activity of PSs, necessitating further refinement to minimize phototoxicity and enhance the performance of PS‐loaded MSNs.

Activatable photosensitizers (aPSs) that exhibit photodynamic activity under specific conditions, such as the presence of specific tumor‐related enzymes, acidic pH, and nucleic acids, reduce PS phototoxicity.^[^ ^9^ ^]^ These aPSs, activated by disease biomarkers, circumvent the phototoxicity of conventional PSs. However, isolated aPSs lack targeted delivery and integration with other therapies. Combining aPSs with nanocarriers, such as MSNs, leverages the advantages of both aPSs and MSNs, enhancing application prospects.^[^ ^10^ ^]^ Nonetheless, owing to the absence of standardized chemical modification strategies, aPSs are physically incorporated into MSNs, leading to variable aPS levels and inconsistent construct reproducibility. Such variability impedes simultaneous drug delivery and the integration of PDT with other treatment modalities. Using modified aPSs as the nanocarrier building blocks enables the synthesis of covalently linked multifunctional nanocarriers.^[^ ^11^ ^]^ This approach maintains aPS functionality and nanocarrier integrity, promising substantial applications. However, owing to a lack of synthesis strategies, such MSN constructs remain unreported.

**Scheme 1:**
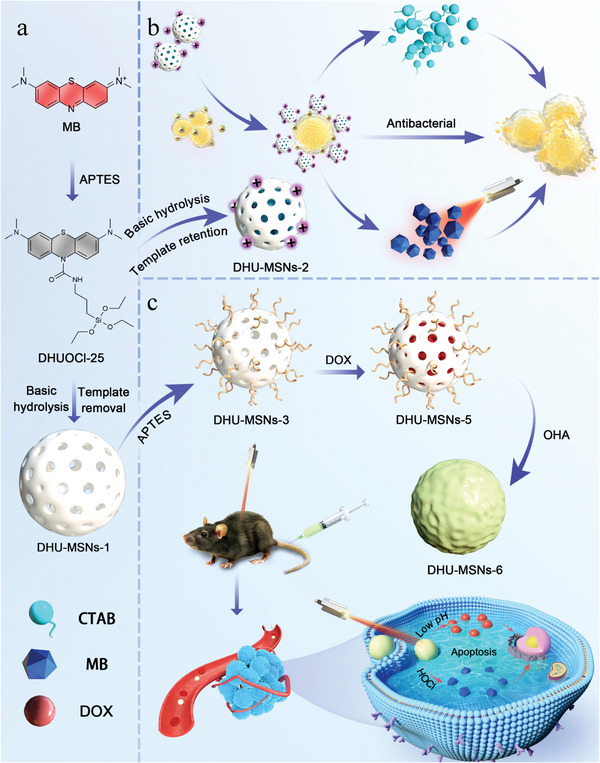
a) Synthesis scheme for the aPS (DHUOCl‐25) and aPS‐covalently modified MSNs (DHU–MSNs‐1). b) Synthesis of DHU–MSNs‐2 and a schematic of its combined photodynamic and antibacterial properties. c) Synthesis of DHU–MSNs‐6 and a schematic of its chemo–photodynamic therapeutic process.

To address this challenge, we investigated the basic building units of MSNs. MSNs are typically synthesized using surfactants as templates via the sol–gel method. Determining the appropriate silica source is crucial for synthesizing MSNs.^[^ ^12^ ^]^ We synthesized a novel silica source, DHUOCl‐25, by improving the conventional silica source, 3‐aminopropyltriethoxysilane (APTES) (**Scheme** **1**a). DHUOCl‐25 functioned as a silica source and PS, releasing the food and drug administration‐approved PS methylene blue (MB) upon treatment with hypochlorous acid (HOCl), an ideal aPS activator. Using DHUOCl‐25, we established a standardized protocol for the covalent modification of MSNs. We synthesized DHU–MSNs‐1, ≈210 nm in diameter, which replicated the behavior of DHUOCl‐25 toward HOCl. We then assessed the newly synthesized aPS for antibacterial and anticancer efficacy. Modifying the synthesis method slightly, we synthesized DHU–MSNs‐2, retaining cetyltrimethylammonium bromide (CTAB) and achieving synergistic antibacterial action via MB PDT and CTAB (antibacterial agent) (Scheme 1b). Confirming that the nano‐aPS retained the properties of MSNs, we converted DHU–MSNs‐1 into DHU–MSNs‐6, which could be loaded with doxorubicin (DOX) (Scheme 1c). Leveraging the drug‐loading capability of MSNs, DHU–MSNs‐6 exhibited exceptional biocompatibility, drug‐loading efficiency, and physiological stability. Cellular experiments revealed that DHU–MSNs‐6 were efficiently internalized and exhibited strong cytotoxicity against the human non‐small cell lung cancer cells (A549 cells). In a murine model of mouse Lewis lung carcinoma (LLC) cells with spinal metastases, DHU–MSNs‐6 demonstrated targeted drug accumulation at tumor sites, with combined chemotherapy and PDT yielding notable anticancer effects. These findings underscore the broad applicability of our strategy for synthesizing activatable mesoporous silica‐based PSs, expanding their potential application.

## 2 Results and Discussion

### 2.1 Design and Characterization of DHUOCl‐25

**Figure 1:**
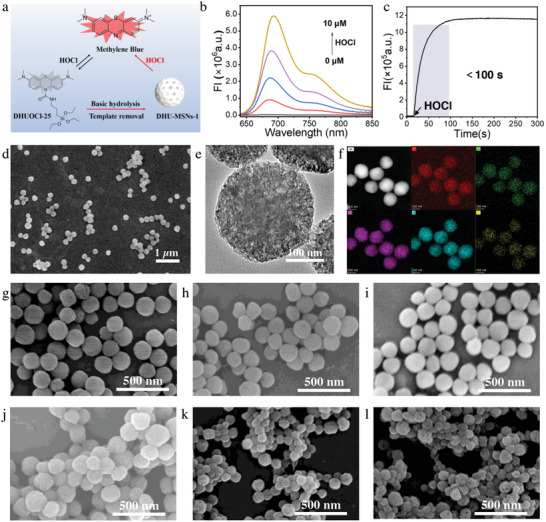
a) Design and working diagram of DHU–MSNs‐1. b) Fluorescence spectra of DHUOCl‐25 (10 µm) before and after the addition of increasing concentrations of HOCl (0, 1, 2, 5, and 10 µm). c) Changes in time‐dependent fluorescence intensity of DHUOCl‐25 (10 µm) at 686 nm upon addition of 10 µm HOCl. d) Scanning electron microscopy (SEM) and e) transmission electron microscopy (TEM) images of DHU–MSNs‐1. f) Energy‐dispersive X‐ray spectroscopy (EDS) analysis of DHU–MSNs‐1 showing the elemental composition (C, N, O, Si, and S). SEM images of g) MSNs, h) DHU–MSNs‐1, and i) DHU–MSNs‐1 + HOCl in PBS at pH 7.4 on day 0. SEM images of j) MSNs, k) DHU–MSNs‐1, and l) DHU–MSNs‐1 + HOCl on day 3.

During aPS synthesis, controlling the structure of intrinsic PSs proves efficacious. In our prior research, we synthesized aPSs suitable for application under various conditions by modifying MB, demonstrating that MB is an ideal PS for derivatization.^[^ ^13^ ^]^ Precursor modification is commonly used for the structural regulation of MSNs. Thus, by combining the structures of the silica source, APTES, and amide‐modified leucomethylene blue (LMB), we synthesized a novel precursor, DHUOCl‐25 (**Figure** **1**a). The synthesis and characterization details of DHUOCl‐25 are provided in the Supporting Information (Figures S1–S4, Supporting Information). Predictably, DHUOCl‐25 reacted with HOCl in phosphate‐buffered saline (PBS, 1.6% ethanol as a cosolvent, pH 7.4), enhancing the fluorescence intensity of DHUOCl‐25 in the 640–850 nm range (Figure 1b). Correspondingly, the absorption of DHUOCl‐25 at 664 nm increased after the addition of HOCl (>307‐fold, Figure S5a, Supporting Information), transitioning from a transparent and colorless solution to a blue solution. The reaction mechanism of DHUOCl‐25 with HOCl aligns with that of previously reported compounds.^[^ ^14^ ^]^ The reaction reached a plateau within 100 s (Figure 1c) and released MB (as demonstrated using liquid chromatography–mass spectrometry (LC–MS) analysis, Figure S5e, Supporting Information), indicating the exceptional sensitivity of DHUOCl‐25. These findings confirm the effective response of DHUOCl‐25 (aPS) toward HOCl. We investigated the selectivity of DHUOCl‐25 toward different amino acids, anions and cations, and ROS. The results, as shown in Figure S5b–d (Supporting Information), revealed no significant changes in the fluorescence upon treatment with 400 µm amino acids (Phe, Trp, Ala, His, Val, Tyr, Thr, Glu, Ser, Leu, Pro, Lys, Arg, Asp, and Gly), anions and cations (CH_3_COO^−^, NH_4_^+^, K^+^, SO_4_^2−^, F^−^, Mg^2+^, NO_2_^−^, ClO_4_^−^, CO_3_^2−^, and Ca^2+^), and 20 µm ROS (H_2_O_2_, ·OH, t‐butyl hydroperoxide (TBHP), ROO·, NO, O_2_^·−^, t‐BuOO·, and ONOO^−^), indicating nonreactivity. However, the fluorescence significantly increased in the presence of HOCl, demonstrating the exceptional selectivity of DHUOCl‐25 toward HOCl.

### 2.2 Morphology and Structure Analysis of DHU–MSNs‐1

Leveraging the exceptional performance of DHUOCl‐25 in aqueous solutions, we proposed its use as a novel silica source to synthesize covalently linked aPS‐MSN. We devised a simple synthesis protocol for aPS‐covalently modified MSNs, yielding DHU–MSNs‐1, a novel mesoporous silica‐based carrier, by optimizing the CTAB content, precursor ratios (TEOS and DHUOCl‐25), alkali concentration, and reaction time. The optimization details and discussion are provided Figure S6a–j (Supporting Information). Scanning electron microscopy (SEM) and transmission electron microscopy (TEM) images revealed the spherical morphology of DHU–MSNs‐1 with diameters of ≈210 nm (Figure 1d,e). The X‐ray diffraction (XRD) pattern exhibited a diffraction peak at 2θ = 22.9° (Figure S7a, Supporting Information), indicating that DHU–MSNs‐1 was composed of amorphous silica. The N_2_ adsorption isotherm exhibited a distinct hysteresis loop, corresponding to the Type IV adsorption isotherm of mesoporous structures (Figure S7b, Supporting Information). The specific surface area of DHU–MSNs‐1 was 107.496 m^2^ g^−1^, as determined using the Barrett–Joyner–Halenda (BJH) model, with a pore volume and diameter of 0.502 cm^3^ and 4.308 nm, respectively. Figure 1f shows the uniform distribution of C (56.31%), N (2.66%), O (27.86%), Si (12.70%), and S (0.47%) in DHU–MSNs‐1. The presence of S signal confirmed the synthesis of DHUOCl‐25‐based MSN. In addition, thermogravimetric analysis confirmed that MB accounted for 10.78% of the total mass of DHU–MSNs‐1 (Figure S8, Supporting Information).

Post synthesis, the response of DHU–MSNs‐1 in an aqueous solution was probed. As shown in Figure S9a–e (Supporting Information), similar to DHUOCl‐25, DHU–MSNs‐1 exhibited good selectivity and sensitivity toward HOCl, with MB liberated upon activation (LC–MS, Figure S9f, Supporting Information). Using the standard curve for MB via UV–vis spectra, we calculated the concentration of MB released by DHU‐MSNs‐1 (100 µg mL^−1^) in response to HOCl (20 µm) being 2.32 µg mL^−1^ (Figure S10, Supporting Information). As could be seen from Figure S11 (Supporting Information), the released MB would be separated from the MSNs. Previous reports indicate that precursor amination accelerates MSN degradation. According to the reaction mechanism of DHU–MSNs‐1 (Figure 1a), amino groups can be released upon activation with HOCl. Therefore, we investigated the degradation behavior of DHU–MSNs‐1. DHU–MSNs‐1 exhibited a slightly accelerated degradation rate than nonfunctionalized MSNs, with further acceleration after the release of MB (Figure 1g–l). This finding indicates that when used as a drug carrier, DHU–MSNs‐1 has an increased potential for rapid drug release upon activation. The synthesis of DHU–MSNs‐1 and its excellent performance confirmed the feasibility of synthesizing aPS‐covalently modified MSNs using aPS as the fundamental building unit.

### 2.3 Synthesis of DHU–MSNs‐2 and its Antibacterial Performance

**Figure 2:**
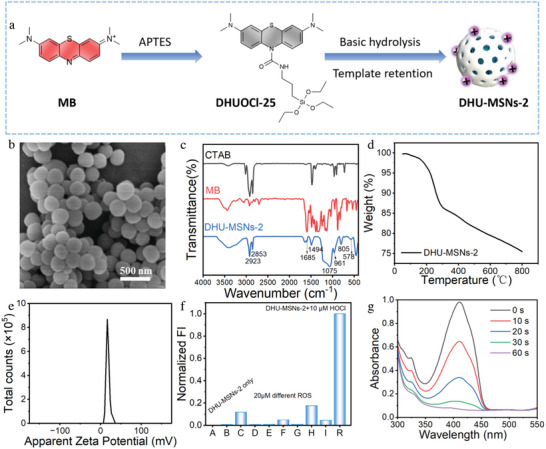
a) Synthetic process for DHU–MSNs‐2. b) SEM image of DHU–MSNs‐2. c) Fourier transform infrared (FTIR) spectra of DHU–MSNs‐2. d) Thermogravimetric curve of DHU‐MSNs‐2. e) Zeta potential of DHU‐MSNs‐2. f) Fluorescence intensity of DHU–MSNs‐2 (20 µg mL^−1^) at 686 nm after treatment with HOCl (10 µm) and various reactive oxygen species (ROS, 20 µm) (B: H_2_O_2_, C: ·OH, D: TBHP, E: ROO·, F: NO, G: O_2_^·−^, H: t‐BuOO·, and I: ONOO^−^). g) Time‐dependent absorption spectra of DPBF upon irradiation with a 658 nm laser (0.3 W cm^−2^) in the presence of DHU–MSNs‐2 and HOCl.

The antibacterial application of PSs is notable. CTAB, a cationic surfactant commonly used in the synthesis of MSN and an antibacterial agent, induces bacterial superoxide stress. Therefore, we modified the synthesis protocol for DHU–MSNs‐1, retaining CTAB, to synthesize DHU–MSNs‐2 for antibacterial applications (**Figure** **2**a). The SEM and TEM images of DHU–MSNs‐2 (Figure 2b; Figure S12a, Supporting Information) exhibited a uniform particle size with a well‐defined mesoporous structure. The spherical particles exhibited diameters of ≈210 nm. The porous structure of DHU–MSNs‐2 was analyzed using N_2_ adsorption–desorption isotherms, which exhibited the characteristic Type IV adsorption isotherm with a distinct hysteresis loop, indicating the presence of a mesoporous structure (Figure S12b, Supporting Information). The absence of a well‐defined saturation adsorption plateau in the hysteresis loop indicated an irregular pore structure, which was consistent with the XRD results (Figure S12c, Supporting Information). Based on the BJH model, the specific surface area of DHU–MSNs‐2 was 33.772 m^2^ g^−1^, with a pore volume of 0.237 cm^3^ and an average pore diameter of 3.412 nm. These results confirmed the successful synthesis of DHU–MSNs‐2. The presence of voids and mesopores in DHU–MSN‐2 facilitated a rapid response to HOCl, accelerating the release of CTAB while facilitating material degradation and enhancing its antibacterial activity.

To analyze the components of DHU–MSNs‐2, we compared the Fourier transform infrared (FTIR) spectra of CTAB, MB, and DHU–MSNs‐2 (Figure 2c). The FTIR spectrum of DHU–MSNs‐2 revealed peaks at 1072 and 802 cm^−^¹ corresponding to the asymmetric and symmetric stretching vibrations of Si─O─Si, while the peak at 962 cm^−^¹ corresponded to the bending vibration of Si─OH. Additionally, the peaks at 1685 and 1494 cm^−^¹ corresponded to the skeletal vibrations of the benzene ring in MB, while the peak at 578 cm^−^¹ corresponded to the C–S–C skeletal vibration in MB. The FTIR data confirmed the successful modification of DHUOCl‐25 onto MSNs. Furthermore, the FTIR peaks at 2923 and 2853 cm^−^¹ corresponded to the stretching vibrations of ─CH_3_ and ─CH_2_─ in the long alkyl chain of CTAB, indicating the successful retention of CTAB in the structure. The FTIR analysis confirmed the successful incorporation of both CTAB and MB onto DHU–MSNs‐2.

Energy‐dispersive X‐ray spectroscopy (EDS) analysis confirmed the elemental composition and their uniform distribution in DHU–MSNs‐2. DHU–MSNs‐2 comprised the following elements: C (52.50%), N (2.87%), O (30.37%), Si (13.77%), and S (0.49%) (Figure S12d, Supporting Information). The presence of S indicated covalent bonding between DHUOCl‐25 and MSNs. EDS analysis validated the successful covalent modification with DHUOCl‐25. Thermogravimetric analysis confirmed that MB and CTAB accounted for 5.39% and 11.59% of the total mass of DHU–MSNs‐2, respectively (Figure 2d).

The surface charge of nanoparticles influences antibacterial activity. The size and surface charge of DHU–MSNs‐2 nanoparticles were determined via dynamic light scattering. The results revealed a uniform nanoparticle size with an average hydrodynamic diameter of ≈210 nm (Figure S12e, Supporting Information), which was consistent with the TEM results. The zeta potential of DHU–MSNs‐2 was 26 mV (Figure 2e). Positively charged nanoparticles facilitate strong electrostatic interactions with bacteria, disrupting bacterial cell membranes, causing leakage of intracellular substances, and leading to bacterial apoptosis.

Given that DHU–MSNs‐2 was designed for HOCl‐activated PS release at the site of bacterial infection, we evaluated its response toward HOCl. The responsiveness of the DHU–MSN‐2 toward HOCl was studied in PBS (pH 7.4). Similar to DHUOCl‐25, DHU–MSNs‐2 exhibited good selectivity and sensitivity toward HOCl (Figure 2f; Figure S14a–f, Supporting Information), with MB release upon activation (LC–MS, Figure S14e, Supporting Information). Photodynamic antibacterial therapy relies on PSs that generate singlet oxygen (^1^O_2_) to kill bacteria. Thus, we used diphenylisobenzofuran (DPBF) to monitor ^1^O_2_ generation by monitoring the absorption change at 410 nm and investigating the correlation between time and power density. DHU–MSNs‐2 exhibited strong potential for ^1^O_2_ generation following exposure to HOCl (Figure 2g). We further used ABDA to assess the single oxygen generation capacity of DHU‐MSNs‐2 (Figure S13, Supporting Information). A biocompatibility analysis of DHU–MSNs‐2 using a CCK‐8 assay with the mouse fibroblast cells (L929) and human gingival epithelial cells indicated that when the mass concentration of DHU–MSNs‐2 increased to 10 µg mL^−1^, the cell viability remained above 90.0% over 12 h (Figure S14g; Figure S14h, Supporting Information). These data indicate that DHU–MSNs‐2 retained the excellent responsive behavior of DHUOCl‐25 toward HOCl, enabling MB release upon activation and exhibiting good biocompatibility.

**Figure 3:**
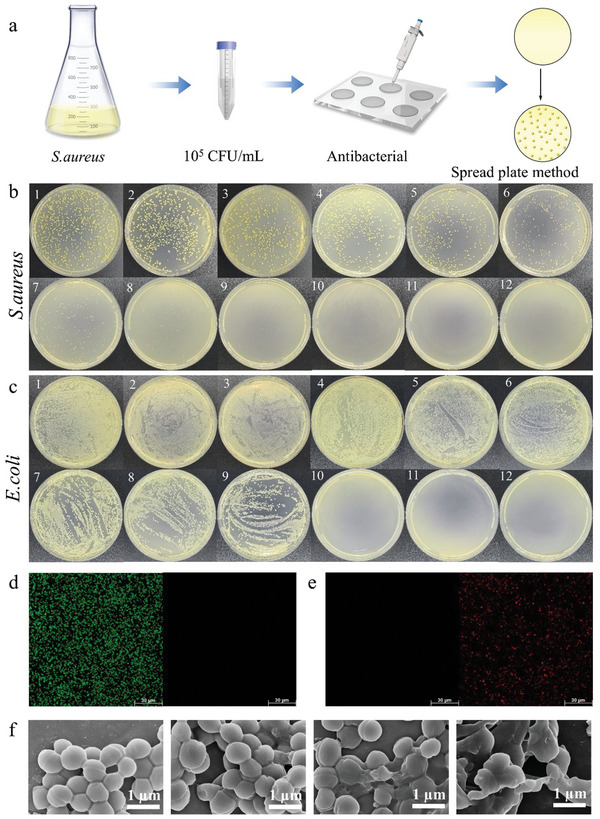
a) Flowchart of the flatbed coating experiment. b) Photographs of the agar plates containing *S. aureus* after various treatments (1. PBS, 2. HOCl (b: 5 µm and c: 10 µm), 3. Laser, 4. 2.5 µg mL^−1^ DHU‐MSNs‐2, 5. 5 µg mL^−1^ DHU‐MSNs‐2, 6. 10 µg mL^−1^ DHU‐MSsNs‐2, 7. 2.5 µg mL^−1^ DHU‐MSNs‐2 + HOCl, 8. 5 µg mL^−1^ DHU‐MSNs‐2 + HOCl, 9. 10 µg mL^−1^ DHU‐MSNs‐2 + HOCl, 10. 2.5 µg mL^−1^ DHU‐MSNs‐2 + HOCl + laser, 11. 5 µg mL^−1^ DHU‐MSNs‐2 + HOCl + laser, and 12. 10 µg mL^−1^ DHU‐MSNs‐2 + HOCl + laser). c) Photographs of agar plates containing *E. coli* after various treatments following the same scheme as in (b). Live or dead fluorescence images of *S. aureus* treated with d) PBS and e) DHU‐MSNs‐2 + HOCl + laser. f) SEM images showing the bacterial morphology of *S. aureus* after various treatments (from left to right: PBS, DHU‐MSNs‐2, DHU‐MSNs‐2 + HOCl, and DHU‐MSNs‐2 + HOCl + laser).

The antibacterial potential of DHU–MSNs‐2 in vitro was evaluated using *Staphylococcus aureus* (*S. aureus*) and *Escherichia coli* (*E. coli*, **Figure** **3**a). The experimental groups were: 1. PBS; 2. HOCl (b: 5 µm and c: 10 µm); 3. Laser; 4. DHU–MSNs‐2 (2.5 µg mL^−1^); 5. DHU–MSNs‐2 (5 µg mL^−1^); 6. DHU–MSNs‐2 (10 µg mL^−1^); 7. DHU–MSNs‐2 (2.5 µg mL^−1^) + HOCl; 8. DHU–MSNs‐2 (5 µg mL^−1^) + HOCl; 9. DHU–MSNs‐2 (10 µg mL^−1^) + HOCl; 10. DHU–MSNs‐2 (2.5 µg mL^−1^) + HOCl + laser; 11. DHU–MSNs‐2 (5 µg mL^−1^) + HOCl + laser; and 12. DHU–MSNs‐2 (10 µg mL^−1^) + HOCl + laser. Neither the HOCl nor the laser group exhibited a significant reduction in bacterial activity compared to the control group (Figure 3b,c). *S. aureus* activity decreased with increasing DHU–MSNs‐2 concentrations (2.5, 5, and 10 µg mL^−1^) compared to the blank owing to the higher concentration of CTAB released at higher DHU–MSNs‐2 concentrations, enhancing the antibacterial effect (Figure 3b). *E. coli* activity also decreased significantly; however, it did not exhibit a concentration‐dependent trend. DHU–MSNs‐2 required a higher concentration of HOCl (10 µm) to significantly reduce the *E. coli* survival rate because gram‐negative bacteria have an outer membrane, making them harder to kill than gram‐positive bacteria. Additionally, the combined effects of PDT and CTAB significantly enhanced the antibacterial effect (Figure 3c).

The validity of the plate‐counting method was confirmed using a staining test. The bacteria in the PBS group emitted green fluorescence, indicating an intact bacterial membrane, whereas increased red fluorescence in the DHU–MSNs‐2 group indicated the inhibitory effect of CTAB (Figure 3d,e; Figure S15a–d, Supporting Information). Higher red fluorescence in the DHU–MSNs‐2 + HOCl group compared to the DHU–MSNs‐2 group indicated that response to HOCl promoted CTAB release and enhanced bacterial inhibition. After DHU–MSNs‐2 + HOCl + laser treatment, the bacteria exhibited red fluorescence, demonstrating the combined antibacterial effect of CTAB and PDT; the combined effect caused severe bacterial cell membrane damage. Consequently, bacterial growth and proliferation were strongly inhibited. Morphological changes in the bacteria were analyzed using SEM. The *S. aureus* in the control group exhibited normal morphology with smooth and intact cell membranes (Figure 3f). However, when treated with DHU–MSNs‐2, *S. aureus* exhibited partial depression and some ruptured membranes. Compared to the control group, the *S. aureus* treated with DHU–MSNs‐2 + HOCl group exhibited significant membrane rupture. In the CTAB and laser group, bacteria collapsed, with ruptured membranes losing their original form. The performance of DHU–MSNs‐2 validated our strategy to synthesize aPS‐covalently modified MSNs using DHUOCl‐25, proving the antibacterial potential of the newly synthesized aPSs.

### 2.4 Synthesis of DHU–MSNs‐6 and its Anticancer Performance

**Figure 4:**
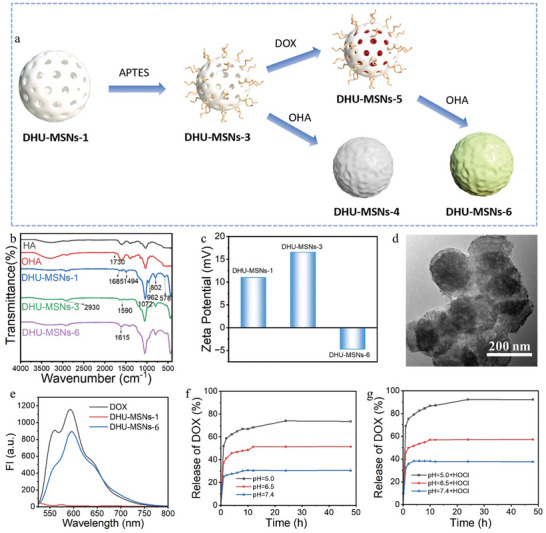
a) Synthesis process for DHU–MSNs‐4 and DHU–MSNs‐6. b) FTIR spectra of hyaluronic acid (HA), OHA, DHU–MSNs‐1, DHU–MSNs‐3, and DHU–MSNs‐6. c) Zeta potential changes of DHU–MSNs‐1, DHU–MSNs‐3, and DHU–MSNs‐6. d) TEM images of DHU–MSNs‐6. e) Fluorescence intensities of H_2_O and doxorubicin (DOX), DHU–MSNs‐1, and DHU–MSNs‐6 in water (excitation wavelength: 480 nm; emission scan range: 525–800 nm). f) DOX release profiles from DHU–MSNs‐6 under different pH conditions (7.4, 6.5, and 5.0). g) DOX release profiles from DHU–MSNs‐6 under different pH conditions (7.4, 6.5, and 5.0) in the presence of HOCl.

Tumor treatment presents a significant application of aPSs. Novel tumor therapy strategies are devised by combining PSs with other treatment modalities. To ensure that aPS‐covalently modified MSNs retained the intrinsic properties of the nanomaterial, we synthesized DHU–MSNs‐6 to integrate chemotherapy and PDT functionalities, building on DHU–MSNs‐1. The synthesis of DHU–MSNs‐6 involved MSN carrier synthesis, surface amination, DOX loading, and hyaluronic acid (HA) modification (**Figure** **4**a). FTIR spectroscopy was used to monitor each reaction step (Figure 4b). Thermogravimetric analysis confirmed that MB accounted for 2.77%, 3.94%, and, 4.88% of the total mass of DHU–MSNs‐3, DHU‐MSNs‐4, and DHU‐MSNs‐6, respectively (Figure S16, Supporting Information).

HA comprises D‐glucuronic acid (GlcA) and N‐acetylglucosamine (GlcNAc). Oxidation by NaIO_4_ converted the two adjacent hydroxyl groups on GlcA of HA into aldehyde groups. The C═O stretching vibration at 1730 cm−^1^ confirmed aldehyde formation in oxidized hyaluronic acid (OHA) (Figure 4b). The skeletal vibrations of the benzene ring at 1685 and 1494 cm^−1^ and C─S─C skeletal vibration at 578 cm^−1^ indicated successful incorporation of FDOCl‐25 onto the MSNs. The asymmetric and symmetric stretching vibrations of Si─O─Si at 1072 and 802 cm^−1^, respectively, and the bending vibration of Si─OH at 962 cm^−1^ indicated the successful incorporation of MB into the mesoporous silica structure of DHU–MSNs‐6. Compared to DHU–MSNs‐1, the new FTIR peaks of DHU–MSNs‐6 at 1568 cm^−1^ (N─H bending vibration) and 2930 cm^−1^ (stretching vibration of the C─H single bond) indicated the incorporation of methylene groups during the amination process. The new peak at 1615 cm^−1^, attributed to the stretching vibration of the C═N double bond, indicated the Schiff base reaction between the aldehyde group on OHA and amino group on DHU–MSNs‐6, leading to successful HA encapsulation on the nanoparticle surface. The hydrated particle size and PDI of DHU‐MSNs‐6 are smaller than that of DHU‐MSNs‐1 and DHU‐MSNs‐3 that lack of HA, suggesting that HA can effectively improve the water dispersion of the material (Figure S17, Supporting Information). The surface charge of DHU‐MSNs‐1 changed from +10 to +17 mV after amino modification, reversing to negative after DOX loading and HA modification (Figure 4c). The SEM image of DHU‐MSNs‐6 indicated a similar particle size to DHU‐MSNs‐1 (Figure S18, Supporting Information). The TEM image of DHU–MSNs‐6 showed a blurred surface structure post‐HA modification, with a uniform coating around the external pores, indicating the successful synthesis steps (Figure 4d).

The characteristic absorption of DOX at 480 nm and its strong fluorescence emission upon excitation at 480 nm were studied using UV–vis absorption and fluorescence spectroscopy, respectively, to confirm the successful loading of DOX. Both DOX and DHU–MSNs‐6 exhibited an absorption peak at 480 nm, which was absent for DHU–MSNs‐1 (Figure S19a, Supporting Information). Additionally, intense fluorescence emission at 590 nm was observed for both DOX and DHU–MSNs‐6 (Figure 4e), whereas this peak was not observed for DHU–MSNs‐1. These results confirmed the successful loading of DOX onto DHU–MSNs‐6. Using the standard curve from the UV–vis absorption spectrum of DOX (Figure S19b, Supporting Information), the drug‐loading and encapsulation efficiencies of DOX on DHU–MSNs‐6 were calculated as 24.11% and 63.55%, respectively.

DHU–MSNs‐6 demonstrated good selectivity and sensitivity toward HOCl, similar to DHUOCl‐25 (Figure S20a–e, Supporting Information), and significant ^1^O_2_ generation in response to HOCl detected by both ABDA (Figure S21, Supporting Information) and DPBF (Figure S22a, Supporting Information). Per our devised scheme, DHU–MSNs‐6 were delivered to tumor sites via blood circulation and responded to HOCl in the tumor microenvironment to release MB. Additionally, in the slightly acidic tumor environment, HA detached from the surface of DHU–MSNs‐6, facilitating DOX release. The color change of DHU–MSNs‐6 in the PB solution (Figure S22b,c, Supporting Information) indicated that MB release was controlled by HOCl. We evaluated the drug release behavior of DHU–MSNs‐6 in vitro by immersing the samples in buffers of different pH values. The results demonstrated that the DOX release rate increased with decreasing pH (5.0, 6.5, and 7.4) (Figure 4f,g). After 48 h, DOX release efficiency was less than 30% at pH 7.4, while it reached 51% and 73% at pH 6.5 and 5.0, respectively. This finding can be attributed to the instability of the imine bonds in DHU–MSNs‐6 under mildly acidic conditions, facilitating drug release. Following HOCl addition, the DOX release efficiency at pH 7.4 remained 30% after 48 h, while it increased to 57% and 92% at pH 6.5 and 5.0, respectively. This finding indicated that HOCl accelerated mesoporous silica degradation and DOX release.

Intravenous administration in mice necessitates nanocomposite stability in the bloodstream. After co‐incubation with a 0.9% NaCl solution (negative control), the mixture remained stratified, with minimal changes in absorbance compared to the baseline (Figure S23a,b, Supporting Information), and no visible red blood cell sedimentation. Increasing DHU‐MSNs‐6 concentration did not significantly affect these results. However, mixing red blood cells with water (positive control) resulted in a uniformly blood‐red solution, with a significant increase in absorbance values. Water causes red blood cells to rupture via osmosis, releasing large amounts of hemoglobin and leading to the observed color change and increased absorbance. These results demonstrated excellent blood compatibility of DHU–MSNs‐6 even at 500 µg mL^−1^. No significant red blood cell rupture was observed (Figure S23a,b, Supporting Information). This finding demonstrates the suitability of DHU–MSNs‐6 for in vivo therapy delivered intravenously to tumor‐bearing mice.

**Figure 5:**
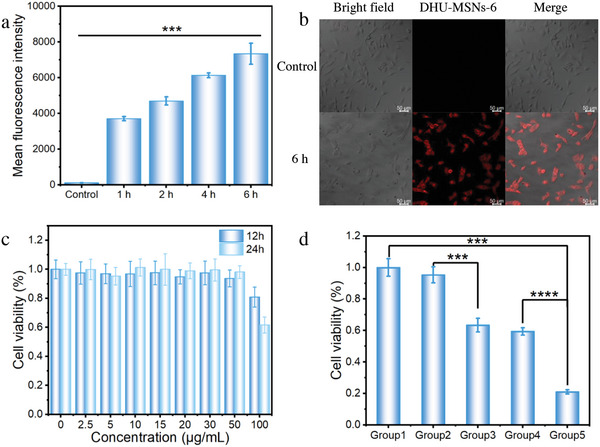
a) Flow cytometry analysis of DHU–MSNs‐6 uptake by the A549 cells. b) Confocal laser scanning microscopy images of the A549 cells treated with DHU–MSNs‐6. c) Cell viability of the A549 cells after 12 and 24 h of incubation with different concentrations of DHU–MSNs‐4. d) Cell viability of the A549 cells after various treatments (Group 1: Control; Group 2: DHU–MSNs‐4 (20 µg mL^−1^); Group 3: DHU–MSNs‐4 (20 µg mL^−1^) + laser (658 nm, 0.3 W cm^−2^, 5 min); Group 4: DHU–MSNs‐6 (15 µg mL^−1^); and Group 5: DHU–MSNs‐6 (10 µg mL^−1^) + laser (658 nm, 0.3 W cm^−2^, 5 min)). ^***^ *p <* 0.001, ^****^ *p <* 0.0001.

Confocal laser scanning microscopy (CLSM) and flow cytometry (FCM) were employed to assess the cellular uptake of DHU–MSNs‐6 by the A549 cells, qualitatively and quantitatively. DOX was used as a fluorescence indicator (excitation wavelength: 480 nm) to demonstrate DHU–MSNs‐6 uptake by the A549 cells. FCM results revealed the time‐dependent uptake of DHU–MSNs‐6 by the A549 cells, increasing over 6 h (**Figure** **5**a). This result was further confirmed by CLSM (Figure 5b), which revealed DOX signals in the cytoplasm of the tumor cells, confirming the successful internalization of DHU–MSNs‐6. These findings indicate that DHU–MSNs‐6 can be rapidly and significantly internalized by the A549 cells, demonstrating their therapeutic potential. Further, co‐incubation of DHU‐MSNs‐6 with cells for different times revealed that the fluorescence of DOX was increasing in the nucleus with increasing incubation time, indicating that the released DOX would enter the nucleus from the cytoplasm (Figure S24, Supporting Information).

DHU‐MSNs‐6 must selectively release MB in tumor cells upon HOCl exposure. While FCM confirms MB release (Figure S25a,b, Supporting Information), it occurs even without external HOCl. Significantly, external HOCl activated higher MB release, potentially minimizing off‐target effects in healthy cells. The effect of different materials on A549 cell viability was evaluated using the CCK‐8 assay. To distinguish MB release from DOX fluorescence (due to overlapping emission), we designed DHU‐MSNs‐4 for specific MB response monitoring (Figure 4a). The key distinction between DHU–MSNs‐4 and DHU–MSNs‐6 was the absence of loaded DOX in DHU–MSNs‐4. Although DHU‐MSNs‐4 exhibited some toxicity at a high concentration under prolonged incubation conditions (Figure S26, Supporting Information), it showed negligible toxicity to A549 cells within a certain concentration range, indicating good biocompatibility (Figure 5c). Figure S27 (Supporting Information) shows a concentration‐dependent negative correlation between DHU–MSNs‐6 and cell viability. In addition, we evaluated the cytotoxicity of different concentrations of CTAB and DOX. CTAB was non‐toxic to cells at low concentrations. However, DOX becomes progressively more cytotoxic with increasing concentration (Figure S28, Supporting Information). The cell viability of the DHU–MSNs‐4, DHU–MSNs‐4 + laser, DHU–MSNs‐6, and DHU‐MSNs‐6 + laser groups was 95.3 ± 5.1%, 63.4 ± 4.3%, 59.4 ± 2.4%, and 21.0 ± 1.3%, respectively, at appropriate material concentrations (Figure 5d). When the concentration of DOX was ≈3 µg mL^−1^, the cell viability of the DOX, DHU‐MSNs‐6, and DHU‐MSNs‐6 + laser groups was 47.89 ± 3.66%, 58.41 ± 1.68%, and 21.01 ± 1.31%. This indicated that the combined photodynamic‐chemotherapy effect promoted the therapeutic efficacy of DOX. The dual staining images of live or dead cells after different treatments confirmed that combined chemotherapy and PDT had significant cytotoxic effects on the A549 cells (Figure S29, Supporting Information).

**Figure 6:**
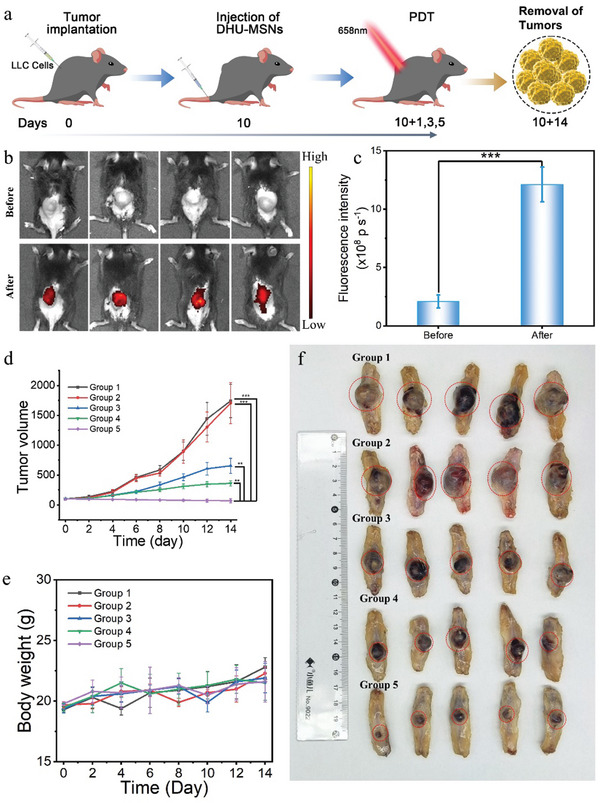
In vivo antitumor activity detection. a) Schematic of the treatment process. b) Fluorescence image of a mouse tumor 24 h after injection. c) Fluorescence image of an isolated tumor. Tumor suppression experiments in hormonal mice. d) Average tumor volume of each group measured over different days. e) Average body weight of mice in each group measured over different days. f) Photograph of the excised tumor from each group after 14 days of treatment. Group 1: Saline; Group 2: DHU–MSNs‐4; Group 3: DHU–MSNs‐4 + laser; Group 4: DHU–MSNs‐6; and Group 5: DHU–MSNs‐6 + laser. ^**p < 0.01, ***^ *p <* 0.001.

Before the in vivo therapeutic experiments (**Figure** **6**a), we confirmed the activation capability of DHU–MSNs‐6 within tumors. We used non‐invasive imaging of MB fluorescence (owing to its intrinsic property) to track DHU‐MSNs‐4 targeting in tumors and HOCl‐activated MB release (Figure 6b). In tumor‐bearing mice, the intravenous injection of DHU–MSNs‐4 resulted in its substantial accumulation in the tumor (Figure 6b,c). This effect can be attributed to the combined effects of nanocomposite size and its specific binding to CD44 receptors via HA. The images show MB release at the tumor site, confirming effective circulation in vivo and minimal toxicity to other healthy tissues.

To evaluate the efficacy of DOX‐loaded DHU–MSNs‐6 in synergizing PDT and chemotherapy in tumor‐bearing mice, we constructed a subcutaneous tumor model of lung cancer by inoculating the LLC cells into the spinal sites of C57 mice. Compared to A549 cells derived from humans, LLC cells derived from mice are more suitable for creating C57 mouse tumor models. Therefore, LLC cells were used for the in vivo experiments. The mice were randomly divided into five treatment groups (*n* = 5): 1) saline, 2) DHU–MSNs‐4, 3) DHU–MSNs‐4 + laser, 4) DHU–MSNs‐6, and 5) DHU–MSNs‐6 + laser. The tumor volumes were recorded to assess the tumor‐inhibitory effect of the materials. After 14 days of treatment, the mice were dissected, and the tumors were excised and photographed. The DHU–MSNs‐4 group exhibited no significant tumor reduction compared to the saline group, indicating that DHU–MSNs‐4 was less toxic to the tumor cells (Figure 6d,e). The DHU–MSNs‐4 + laser group exhibited significant tumor inhibition, demonstrating the antitumor effect of PDT. The DHU–MSNs‐6 group exhibited a notable reduction in tumor size compared to the saline group, indicating effective DOX release in the tumor microenvironment, thus inhibiting tumor cell growth. The DHU–MSNs‐6 + laser group exhibited the greatest tumor volume reduction, indicating enhanced antitumor efficacy from the combined photodynamic and chemotherapy treatment. During the treatment, the body weights of the mice did not fluctuate significantly (Figure 6f), indicating good biocompatibility of DHU–MSNs‐6 in vivo. This study confirmed that DHU–MSNs‐6, synthesized from DHUOCl‐25 using a standardized protocol, retained excellent performance. Furthermore, it enabled the synergistic combination of aPSs with other therapeutic approaches, promising potential applications in tumor therapy.

The physical condition of the treated mice was further evaluated using H&E staining. The morphology of the organs was not significantly changed in all groups after treatment compared to the control group, which suggests that the materials have minimal side effects in vivo (Figure S30, Supporting Information).

## 3 Conclusion

To enhance the performance of existing PSs, we synthesized an aPS (DHUOCl‐25) activatable by HOCl. This aPS also functioned as a silica source, enabling the synthesis of aPS‐covalently modified MSNs (DHU–MSNs‐1) via a standardized protocol. Using the same strategy, DHU–MSNs‐2 were synthesized by retaining CTAB to achieve synergistic antibacterial effects through the combined action of PDT and CTAB. DHU–MSNs‐6, loaded with DOX and modified with HA, were synthesized to confirm the retention of the drug‐loading capacity of the MSNs by the synthesized aPS. Activated by HOCl, DHU–MSNs‐6 released MB, and the low pH of the tumor microenvironment facilitated rapid DOX release. During cellular experiments, DHU–MSNs‐6 was rapidly internalized by the A549 cells, exhibiting strong cytotoxicity. In tumor‐bearing C57BL/6 mice with LLC, DHU–MSNs‐6 accumulated at tumor sites and demonstrated anticancer effects via combined chemotherapy and PDT. These results confirm the broad application prospects of aPS‐covalently modified MSNs, expanding the applications for MSNs and PSs. In summary, our study demonstrates the potential of DHUOCl‐25 and its derived aPS‐covalently modified MSNs (DHU–MSNs) in enhancing the functionality and application range of PSs and MSNs.

## 4 Experimental Section

### Materials

Methylene blue (MB), di(trichloromethyl) carbonate (BTC), sodium carbonate, sodium dithionite, (3‐aminopropyl) triethoxysilane (APTES), tetraethyl silicate (TEOS), cetyltrimethylammonium bromide (CTAB), sodium hydroxide, triethylamine, dichloromethane, ethyl acetate, petroleum ether, and ethanol were of analytical grade and purchased from Shanghai Adamas Reagent Company. AGAR powder and LB broth were sourced from Shenggong Bioengineering (Shanghai) Co., Ltd.

### Preparation of Different Analytes

All solvents used in optical spectroscopic studies were of analytical grade, and ultrapure water was employed for the preparation of analytes. Stock solutions of HOCl (1 mm) were prepared in double‐distilled water (ddH₂O). Other analytes were prepared in ddH₂O following previously reported protocols.^[^ ^15^ ^]^ H_2_O_2_ was diluted from a 30% solution. ·OH (Hydroxyl radical) generated by the Fenton reaction, in which H₂O₂ was added in the presence of 10 equivalents of ferrous chloride. The concentration of ·OH was set equal to that of H₂O₂. The concentration of ·OH was equal to the H_2_O_2_ concentration. TBHP (tert‐butyl hydroperoxide) was obtained from 70% TBHP solution in ddH_2_O. ROO^•^ was prepared by dissolving 2,2′‐azobis(2‐amidinopropane) dihydrochloride in ddH_2_O. t‐BuOO· was prepared by adding TBHP in the presence of 10 equiv. of ferrous chloride. The concentration of t‐BuOO· was equal to the TBHP concentration. O_2_^−^ was prepared by dissolving KO_2_ (potassium superoxide) in DMSO. ONOO^−^ was prepared using 3‐morpholinosydnonimine hydrochloride. HOCl was obtained from 0.1 mol L^−1^ NaOCl solution, this standard solution was purchased from MACKLIN reagent. Stock solutions (5 mm) of other analytes, including Phe, Trp, cys, Ala, His, Val, Tyr, Thr, Glu, Ser, Leu, pro, Lys, Arg, Asp, Gly, CH_3_COO^−^, NH_4_^+^, K^+^, SO_4_^2−^, S_2_O_3_^2−^, F^−^, Mg^2+^, NO_2_^−^, ClO_4_^−^, CO_3_^2−^, Cu^2+^, Ca^2+^, were also prepared in ddH₂O.

### The Synthesis of DHUOCl‐25

FDOCl‐2 was synthesized according to this reported procedure.^[^ ^14b^ ^]^ 5 g (0.5 eq, 14.41 mmol) of FDOCl‐2 and 3 mL of triethylamine were dissolved completely in 30 mL of dry dichloromethane. Under ice bath conditions, 5 mL (0.8 eq, 21.55 mmol) of 3‐aminopropyltriethoxysilane, dissolved in 5 mL of dichloromethane, was added dropwise via a pressure‐equalizing dropping funnel. The mixture was stirred continuously until the reaction reached completion, as confirmed by TLC analysis (≈2.5 h). The organic phase was then dried over anhydrous sodium sulfate, filtered, and concentrated to yield a crude product. The product was purified by column chromatography using a dichloromethane/ethyl acetate (30:1) eluent system, resulting in DHUOCl‐25 as a pale‐yellow oily substance with a yield of 0.8 g (62%). ^1^H NMR(400 MHz, DMSO‐*d_6_*, δ)7.25 (d, *J* = 8.8 Hz, 2H), 6.70 (d, *J* = 2.8 Hz, 2H), 6.67–6.64 (m, 2H), 6.00 (t, *J* = 5.8 Hz, 1H), 3.74–3.69 (m, 6H), 2.99 (dd, *J* = 6.8, 13.2 Hz, 2H), 2.89 (s, 12H), 1.45–1.39 (m, 2H), 1.13 (t, *J* = 7.0 Hz, 9H), 0.48–0.44 (m, 2H). ^13^C NMR (100 MHz, CDCl_3_, δ) 155.8, 148.6, 133.9, 128.3, 127.0, 110.9, 110.6, 58.1, 42.9, 40.4, 23.1, 18.1, and 7.2. Maldi‐tof: [M+H^+^] calcd for C_26_H_41_N_4_O_4_SSi: 533.2613; found: 533.2617.

### Synthesis of DHU‐MSNs‐1

Following a series of optimizations (see Supporting Information), the optimal experimental conditions were established as follows: 500 mg of CTAB was dissolved in a mixture of 42 mL of water and 18 mL of anhydrous ethanol under stirring. Subsequently, 0.3 mL of NaOH solution (2 mol L^−1^) was added at room temperature. After 30 min of hydrolysis, 1.4 mL of TEOS was introduced into the system. Following this, 191 mg of DHUOCl‐25, pre‐dissolved in 1 mL of anhydrous ethanol, was added, and the mixture was stirred continuously at room temperature for 1 h. Upon completion of the reaction, the precipitate was collected by centrifugation, refluxed in anhydrous ethanol, centrifuged again, and freeze‐dried to yield the final product.

### Synthesis of DHU‐MSNs‐2

The synthesis of DHU‐MSNs‐2 followed a protocol similar to that of DHU‐MSNs‐1, with one key modification that CTAB was only partially removed. This was achieved by reducing the number of refluxes and washes during the purification process.

### Antibacterial Activity of DHU‐MSNs‐2

The in vitro antibacterial activity of DHU‐MSNs‐2 was evaluated against *Staphylococcus aureus* (*S. aureus*) and *Escherichia coli* (*E. coli*). Bacterial suspensions (10⁵ CFU mL^−1^) were co‐incubated with varying concentrations of DHU‐MSNs‐2 at 37 °C in a constant‐temperature shaker for 1 h. In the synergistic antibacterial group, a 658 nm laser (0.4 W cm^−^^2^) was applied for 5 min for photodynamic sterilization, followed by continued incubation for an additional 24 h. The bacterial suspensions were then diluted to 10^3^ CFU mL^−1^, and 100 µL of each dilution was spread evenly on agar plates. After incubation at 37 °C for 24 h, live bacterial counts were visualized via agar plate photographs.

### Morphological Characterization of Bacteria

Bacteria treated with DHU‐MSNs‐2 (10 µg mL^−1^) under near‐infrared light (0.4 W cm^−^^2^ for 5 min) were centrifuged at 5000 rpm for 5 min and washed with PBS. The bacterial pellets were then fixed with 2.5% glutaraldehyde at room temperature for 4 h. Subsequently, the fixed samples were dehydrated using a graded ethanol series (30%, 50%, 70%, 90%, and 100%). The dehydrated bacterial samples were suspended in ethanol, deposited onto copper grids, and analyzed via SEM. Bacteria treated with PBS served as controls.

### Synthesis of DHU‐MSNs‐3

DHU‐MSNs‐1 of 100 mg was dispersed in 100 mL of anhydrous ethanol. Subsequently, 1 mL of APTES was added, and the reaction was refluxed at 80 °C for 24 h to obtain DHU‐MSNs‐3.

### Synthesis of DHU‐MSNs‐6

At room temperature, 10 mg of DHU‐MSNs‐3 and 5 mg of adriamycin hydrochloride (DOX) were dissolved in 10 mL of ultrapure water, stirred, and protected from light for 24 h. The resulting precipitates were centrifuged, washed three times with ultrapure water, and the supernatants were collected for UV‐vis spectroscopic analysis. The precipitates were then redispersed in 10 mL of ultrapure water, followed by the addition of 10 mg of OHA. The mixture was stirred at room temperature under light protection for 12 h. The precipitates were centrifuged, collected, and freeze‐dried to yield DHU‐MSNs‐6 nanoparticles. The product was characterized by FTIR spectrometer, zeta‐potential and particle size analyzer, TEM, UV–vis absorption, and fluorescence spectrophotometer.

### Variants of DHU‐MSNs‐6

A similar method was employed to synthesize DOX‐free but HA‐encapsulated DHU‐MSNs‐4. The intermediate material containing DOX but lacking HA encapsulation was denoted as DHU‐MSNs‐5.

### Drug Loading and Encapsulation Efficiency

The DOX content in DHU‐MSNs‐6, characterized by a distinct UV absorption peak at 480 nm, allowed indirect calculation of drug loading (%) and encapsulation (%) using a UV–vis spectrophotometer to measure the absorbance of the supernatant collected after centrifugation. The following equations were applied:

(1) $$\begin{array}{c} \mathrm{Loading}\;\mathrm{efficiency}\left(\%\right)=\left[\left(M_{0}-M_{1}\right)/\left(M_{2}+M_{0}-M_{1}\right)\right]\times 100\% \end{array}$$

(2) $$\mathrm{Entrapment}\;\mathrm{efficiency}=[\left(M_{0}-M_{1}\right)/M_{0}]\times 100\%$$

where M_0_ is the amount of initial DOX, M_1_ is the amount of unloaded DOX, M_2_ is the amount of nanocarrier.

### In Vitro Responsive Drug Release of DHU‐MSNs‐6

The drug release behavior of DHU‐MSNs‐6 was investigated in PBS at pH values of 5.0, 6.5, and 7.4. Samples (1 mL) were prepared in 1.5 mL centrifuge tubes and incubated on a shaker at 100 rpm. At predetermined time points (1, 2, 4, 6, 8, 10, 12, 24, and 48 h), individual tubes were removed and centrifuged. The supernatant was collected, and its absorbance at 480 nm was measured using a UV–vis spectrophotometer. The amount of DOX released was calculated based on a standard curve of DOX.

### Nanocomposite Response in Tumor Cells

A549 cells were seeded into 6‐well plates at a density of 2 × 10^5^ cells/well and cultured overnight. The cells were divided into five groups: Control, DHU‐MSNs‐4, DHU‐MSNs‐4 + HOCl, DHU‐MSNs‐6, and DHU‐MSNs‐6 + HOCl. Fresh medium containing the corresponding materials was added to each group and incubated for 6 h. After incubation, the medium was removed, and cells were washed three times with PBS. Subsequently, 2 mL of 30 µm HOCl solution was added and incubated for 10 min. Following this treatment, the HOCl solution was removed, and the cells were washed three times with PBS. The cells were collected by trypsinization, and fluorescence intensity was analyzed using flow cytometry.

### Cytotoxicity Assays

The cytotoxicity of DHU‐MSNs‐4 and DHU‐MSNs‐6 was evaluated using human non‐small cell lung cancer A549 cells. Cells were cultured in DMEM at 37 °C in a 5% CO₂ incubator and seeded into 96‐well plates at a density of 1 × 10^4^ cells/well and incubated overnight. The cells were treated with various concentrations of DHU‐MSNs‐4 and DHU‐MSNs‐6 for 24 h. After incubation, 100 µL of CCK‐8 reagent (diluted with basal medium) was added to each well and incubated for 1 h. Absorbance at 450 nm was recorded using a microplate reader. For PDT experiments, cells were divided into five groups: Control, DHU‐MSNs‐4, DHU‐MSNs‐4 + Laser, DHU‐MSNs‐6, and DHU‐MSNs‐6 + Laser. After adding materials, the PDT groups were irradiated with a 658 nm laser (300 mW cm^−^^2^) for 5 min, while the Control group was shielded from light. Following laser irradiation, the cells were incubated for an additional 24 h. The CCK‐8 assay was used to determine cell viability.

### In Vivo Fluorescence Imaging

All animal experiments were approved by the Research Ethics Committee of Zhongshan Hospital, Fudan University (2020‐032). The biodistribution of DHU‐MSNs‐4 was evaluated via fluorescence imaging in a mouse spinal subcutaneous tumor model. Tumor‐bearing mice were injected via the tail vein with 100 µL of DHU‐MSNs‐4 solution (5 mg mL^−1^, 𝑛 = 4). At 24 h post‐injection, mice were anesthetized with isoflurane, and in vivo, fluorescence imaging was performed using excitation and emission wavelengths of 660 and 710 nm, respectively. Following imaging, the mice were euthanized, and tumors were excised for ex vivo fluorescence imaging. The average fluorescence intensity of the tumor regions was quantified using the associated imaging software.

### In Vivo Chemotherapy‐Photodynamic Combination Therapy

The therapeutic efficacy of DHU‐MSNs‐4 and DHU‐MSNs‐6 was assessed in C57BL/6 mice with subcutaneous tumors (≈100 mm^3^). Mice were randomly assigned to five groups: 1) PBS, 2) DHU‐MSNs‐4, 3) DHU‐MSNs‐4 + Laser, 4) DHU‐MSNs‐6, and 5) DHU‐MSNs‐6 + Laser. Each mouse was administered 100 µL of PBS containing the respective material via tail vein injection. For laser treatment, tumor sites in the light groups were irradiated with a 658 nm NIR laser (500 mW cm^−^^2^) for 30 min at 24 h post‐injection, with additional irradiation on days 3 and 5. Tumor volumes and body weights were recorded every 2 days for 14 days. Relative tumor volumes were calculated as 𝑉/𝑉_0_, where 𝑉_0_ is the initial tumor volume. Tumor volume (𝑉) was determined using the formula: 𝑉 = (𝐿•𝑊^2^)/2 where𝐿 is the maximum tumor length and W is the minimum tumor width, measured with calipers. At the end of the study, tumors were excised and measured.

### Statistical Analysis

Data were analyzed using GraphPad Prism 8 software and presented as mean ± standard deviation. Statistical significance was assessed using two‐tailed P‐values, with thresholds set as follows: ^*^ *p*<0.05, ^**^ *p*<0.01, ^***^ *p*<0.001, ^****^ *p*<0.0001.

## Conflict of Interest

The authors declare no conflict of interest.

## Supporting information



Supporting Information

## Data Availability

The data that support the findings of this study are available in the supplementary material of this article.
