# Supplementary material for: Constructing Activatable Photosensitizers Using Covalently Modified Mesoporous Silica
Source: Adv Sci (Weinh). 2025 Jan 15;12(9):2406887. doi: 10.1002/advs.202406887 (PMC11884586; doi:10.1002/advs.202406887)
Supplement: Supplementary file 1 — Supporting Information [file ADVS-12-2406887-s001.docx]

**Supporting information**

Constructing Activatable Photosensitizers Using Covalently Modified Mesoporous Silica

Yan Liu^1 #^, Xiang Wang^1#^, Ben Wang^2^, Zhenni Lu^1^, Changru Wu^1^, Zhanghao He^1^, Libo Jiang^2*^, Peng Wei^1*^, and Tao Yi^1*^

Y. Liu, X. Wang, Z. Lu, C. Wu, Z. He, Prof. P. Wei, Prof. T. Yi

State Key Laboratory for Modification of Chemical Fibers and Polymer Materials, College of Chemistry and Chemical Engineering, Donghua University, Shanghai 201620, China

E-mail: weipeng@dhu.edu.cn; yitao@dhu.edu.cn.

Dr. B. Wang, Prof. L. Jiang

Department of Orthopaedic Surgery, Zhongshan Hospital, Fudan University, Shanghai 200032, China

Dr. B. Wang have moved to Orthopedic Department, Taizhou Hospital of Zhejiang Province，Taizhou, Zhejiang 317000, China

[libo@zs-hospital.sh.cn](mailto:libo@zs-hospital.sh.cn)

^#^Y. Liu, and X. Wang contributed equally to this work.

# 1. Instruments

^1^H NMR (400 MHz) and ^13^C NMR (100 MHz) spectra were taken on a AVANCE NEO 400 M nuclear magnetic resonance spectrometer (Bruker Company, Switzerland), using CD_3_Cl or DMSO-*d*_6_ as solvent. Proton or carbon chemical shifts are reported in parts per million downfield from tetramethylsilane (TMS), with TMS (δ = 0.0 ppm) or the solvent residue peak CD_3_Cl (77.16 ppm for ^13^C) as the chemical shift standard. Maldi-tof was measured on AB SCIEX 5800 MALDI TOF/TOF™. UV-visible spectra were recorded on a Shimadzu UV-2600 spectrometer. Steady-state fluorescent spectra at room temperature were measured on an Edinburgh instrument FLS-1000 spectrometer with a Xe lamp as an excitation source. Confocal laser scanning microscopy (CLSM) images were obtained with Leica SP8. In vivo images were collected using a IVIS Lumina Ⅲ small animal in vivo fluorescence imaging system. The transmission electron microscopy (TEM) images were performed on a JEM-2100 field emission transmission electron microscope (Hitachi) with an accelerating voltage of 200 KV. Dynamic light scattering (DLS) and Zeta potential were performed on a Nanor-ZS Zetasizer (Malvern, UK).

**2. Synthesis**

**Figure S1** The specific synthesis process of DHUOCl-25.

The synthetic conditions of DHU-MSNs-1 was described in Table S1.

Table S1 Synthesis conditions of DHU-MSNs-1

| Groups | A：NaOH  （2 mol/L）（mL） | B：TEOS  （mL） | C：DHUOCl-25（mg） | D：Reaction time（h） |
| --- | --- | --- | --- | --- |
| 1 | 0.6 mL | 2.8 mL | 191 mg | 8 h |
| 2 | 0.6 mL | 2.8 mL | 382 mg | 8 h |
| 3 | 0.6 mL | 2.8 mL | 191 mg | 1 h |
| 4 | 0.6 mL | 2.8 mL | 191 mg | 8 h |
| 5 | 0.6 mL | 0.7 mL | 191 mg | 1 h |
| 6 | 0.6 mL | 1.4 mL | 191 mg | 1 h |
| 7 | 0.6 mL | 2.8 mL | 191 mg | 1 h |
| 8 | 0.3 mL | 1.4 mL | 191 mg | 1 h |
| 9 | 0.6 mL | 1.4 mL | 191 mg | 1 h |
| 10 | 1.2 mL | 1.4 mL | 191 mg | 1 h |

DHUOCl-25, composed of methylene blue (MB) and 3-aminopropyltriethoxysilane (APTES), has its MB end uninvolved in the synthesis of mesoporous silica nanoparticles (MSN), with only the APTES end taking part in the reaction. APTES serves as a silane coupling agent and can also act as a silica source. However, its sole use as a silica source typically does not yield precipitation. Hence, in this synthesis, DHUOCl-25 serves as a doped silica source and participates in the reaction alongside tetraethyl orthosilicate (TEOS). The hydrolysis mechanism of TEOS is well-established, but the impact of DHUOCl-25 doping on MSN synthesis remains uncertain. Therefore, we investigated the influence of different amounts of DHUOCl-25 doping on the size and morphology of the nanoparticles. As depicted in Figure S6a-b, when the doping amounts of DHUOCl-25 were 191 mg and 392 mg, the nanoparticles exhibited spherical structures with sizes ranging from 1 to 2 µm. This indicates that the doping amount of DHUOCl-25 has no significant effect on the size and morphology of the nanoparticles. Therefore, considering the conservation of materials, the optimal amount of DHUOCl-25 was determined to be 191 mg.

The reaction time refers to the duration from the completion of CTAB hydrolysis to the end of the reaction after adding the silica source. We investigated the influence of different reaction times on the morphology of the nanoparticles. As shown in Figure S6c-d, when the reaction times were 1 h and 8 h, spherical nanoparticles were formed with sizes around 400 nm and 500 nm, respectively. However, increasing the reaction time led to larger size distributions of the nanoparticles, reducing their uniformity. Therefore, the optimal reaction time was determined to be 1 hour.

The concentration of the silica source can affect its hydrolysis rate, thereby influencing the nucleation rate of nanoparticles. At the optimal reaction time, with TEOS quantities of 0.7 mL, 1.4 mL, and 2.8 mL, as depicted in Figure S6e-g, spherical nanoparticles were observed. Specifically, when 0.7 mL of TEOS was used, the nanoparticles exhibited adhesion and blurred outlines. In contrast, with 1.4 mL and 2.8 mL of TEOS, the nanoparticles were approximately 500 nm in size and uniformly dispersed. Considering cost-effectiveness, the TEOS quantity was selected as 1.4 mL.

The sodium hydroxide (NaOH) solution (2 mol/L) provides an alkaline environment wherein CTAB self-assembles into micelles. The silica precursor molecules then condense on the surface to form silica, and after removal of the surfactant, mesoporous structures are obtained. Therefore, the amount of alkali added is crucial for the synthesis of MSN. As the alkalinity increases, the hydrolysis rate of TEOS gradually accelerates, leading to a higher concentration of silicic acid monomers in the solution and an increased number of nuclei formed. However, with a constant concentration of reactants in the entire system, the nucleation stage is affected, resulting in the formation of smaller and incomplete nanoparticles. We investigated the effect of different amounts of NaOH addition on the nanoparticles. At the optimal reaction time and TEOS quantity, as shown in Figure S6h-j, adding 0.3 mL of NaOH solution resulted in the formation of approximately 200 nm spherical nanoparticles that were uniformly dispersed. However, irregularly shaped nanoparticles were formed when 0.6 mL of NaOH solution was added, and with 1.2 mL of NaOH, uniformly dispersed nanoparticles were not obtained. Therefore, 0.3 mL of NaOH solution was selected.

## 2. Biotoxicity assessment of DHU-MSNs-2

The toxicity of DHU-MSNs-2 was evaluated using mouse fibroblasts (L929) and human gingival epithelial cells (HGE). Both L929 and HGE cells were cultured in DMEM medium at 37℃ with a CO_2_ concentration of 5% in the incubator. L929 and HGE cells were inoculated into 96-well plates at a density of approximately 2×10^5^ cells per well and incubated overnight. Different concentrations of DHU-MSNs-2 were then co-cultured with the cells for 12 or 24 hours. To assess cytotoxicity, 100 µL of CCK-8 (diluted with basal medium) was added to each well, and the cells were then continued to incubate in the incubator for 2-4 hours. Subsequently, the optical density (OD) of each well was measured at 450 nm using an enzyme marker.

## 3. Staining assay

The antibacterial activity of DHU-MSNs-2 was further examined through live/dead assay. Briefly, bacterial suspension (10^8^ CFU/mL, 100 µL) was incubated with DHU-MSNs-2 (10 µg mL^−1^, 100 µL) for 3 h, and after that, the suspension was exposed to NIR light (0.4 W cm^−2^, 5 min). The suspension was centrifuged before staining with PI and DAPI in the dark (30 min), the suspension was washed, and the bacteria were resuspended with PBS. Finally, the suspension was dropped on slides and observed by confocal laser scanning microscopy (CLSM, Leica TCS SP8 X). Bacteria treated with PBS were set as control.

## 4. Synthesis of oxidized hyaluronic acid (OHA)

OHA was prepared by NaIO_4_ oxidation method. 1.00 g (2.49 mmol) of HA and 0.32 g (1.50 mmol) of NaIO_4_ were dissolved in 100 mL of ultrapure water and stirred for 2 h, protected from light, and then the unreacted NaIO_4_ was quenched by the addition of 0.5 mL of ethylene glycol. The reaction solution was dialyzed with ultrapure water (10 kDa) for 3 days and then freeze dried to obtain OHA.

## 5. Hemolysis assay

Take 2 mL of fresh mouse blood, add it to a centrifuge tube containing sodium heparin, dilute it with 0.9% NaCl solution, shake it well, and centrifuge it (3000 r/min, 5 min), and the precipitated erythrocytes were washed with 0.9% NaCl solution until the upper layer of the solution was clarified. DHU-MSNs-6 was added to the collected mouse erythrocytes, and the hemolytic ability of DHU-MSNs-6 was assessed by detecting the absorbance value of erythrocytes. Positive and negative controls were treated with ultrapure water and 0.9% NaCl solution for erythrocytes, respectively. Then, different concentrations of DHU-MSNs-6 (50, 100, 150, 200 and 500 µg mL^−1^) were added (diluted with 0.9% NaC1 solution). The mixture was left to stand at 37℃ for 2 h before being photographed and the absorbance at 540 nm of the supernatants of the seven groups of samples was determined.

$$\mathrm{Hemolysis}\left( \% \right)=\frac{A_{2}-A_{0}}{A_{1}-A_{0}}\times100\%$$

Where A_0_ is the negative control absorbance, A_1_ is the positive control absorbance and A_2_ is the sample absorbance.

## 6. DHU-MSNs-6 cell uptake assay

First, the endocytosis performance of A549 cells towards the synthesized nanoparticles DHU-MSNs-6 was quantified by flow cytometry (FCM). A549 cells were inoculated in 6-well plates at a density of 2×10^5^ cells/well, and then incubated overnight at 37℃. After removing the original medium, fresh medium containing DHU-MSNs-6 was added, and the incubation was continued for 1, 2, 4, and 6 h. Then the medium was removed, and the cells were rinsed with PBS three times. After trypsin digestion, the cells were collected and the fluorescence intensity was detected by flow cytometry.

After that, confocal laser scanning microscopy (CLSM) was used to verify the results of flow cytometry. A549 cells were inoculated in glass-bottomed petri dishes at a density of 1×10^5^ cells/well, and then incubated at 37℃ overnight. After removing the original medium, fresh medium containing DHU-MSNs-6 was added and the incubation was continued for 6 h. Then the medium was removed and the incubated cells were rinsed with fresh PBS for three times, and finally, the cells were observed using a confocal fluorescence microscope, and all the images were captured under 480 nm excitation.

## 7. Live/dead cell double staining assay

A549 cells were inoculated in 6-well plates at a density of 6×10^5^ cells/well, after they were allowed to grow adherently to the wall and co-incubated with different materials. 6 groups were set up for this experimental grouping: PBS, Laser, DHU-MSNs-4, DHU-MSNs-4+Laser, DHU-MSNs-6A and DHU-MSNs-6+Laser. the PDT group was irradiated with a 658 nm laser (0.3 W cm^−2^) for 5 min while the control group needed to be protected from light. After co-incubation of the material and cells in the light group for 6 h, the corresponding laser irradiation was given and the incubation was continued for another 24 h. At the end of the incubation, Calcein-AM (1 µL)/Propidium iodide PI (3 µL) was added to the medium and incubated together for another 30 min. Finally, the cells were washed three times with PBS (pH=7.4) and imaged by CLSM imaging.

## 8. Establishment of tumor model

Tumor models were established by subcutaneous injection of 25 µL of PBS solution containing 1×10^6^ LLC cells (mouse lung cancer cells) into the spinal region of C57 female mice. When the tumor volume reached approximately 100 mm^3^, the loaded mice could be used for subsequent experiments, in which the tumor volume was calculated by the following formula: V=L⋅W^2^/2 (L is the maximum length of the tumor, and W is the minimum width of the tumor, both measured by vernier calipers).

# 9. Additional Figures

**Figure S2** ^1^H NMR spectra of DHUOCl-25 in DMSO-*d_6_.*

**Figure S3** ^13^C NMR spectrum of DHUOCl-25 in CDCl_3_.


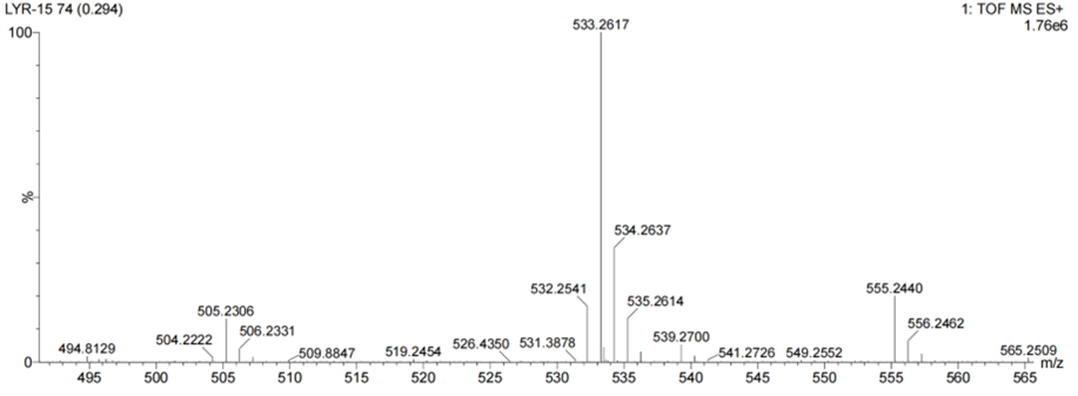


**Figure S4** MALDI-TOF MS of DHUOCl-25 in methanol.

**Figure S5** (a) Absorption spectra of DHUOCl-25 (10 µM) before and after response with 10 µM HOCl. (b) Fluorescence intensity of DHUOCl-25 (10 µM) at 686 nm after treatment with HOCl (10 μM) and amino acids (400 µM) (from B to Q: Phe, Trp, Cys, Ala, His, Val, Tyr, Thr, Glu, Ser, Leu, pro, Lys, Arg, Asp, Gly). (c) Fluorescence intensity of DHUOCl-25 (10 µM) at 686 nm after treatment with HOCl (10 μM) and different ions (400 µM) (from B' to K': CH_3_COO^−^, NH_4_^+^, K^+^, SO_4_^2−^, F^−^, Mg^2+^, NO_2_^−^, ClO_4_^−^, CO_3_^2−^, Ca^2+^). (d) Fluorescence intensity of DHUOCl-25 (10 µM) at 686 nm after treatment with HOCl (10 μM) and ROS (20 µM) (from B'' to I'': H_2_O_2_, ·OH, TBHP, ROO·, NO, O_2_^·−^, t-BuOO·, ONOO^−^). (e) LCMS plot of DHUOCl-25 (10 μM) after response with 10 µM HOCl.

**Figure S6** (a-j) Correspond to SEM images of the prepared materials 1-10 in Table S1, respectively.

**Figure S7** (a) Wide-angle XRD spectra and (b) BET nitrogen adsorption/desorption isotherms (inset: BJH pore size distributions) of DHU-MSNs-1.


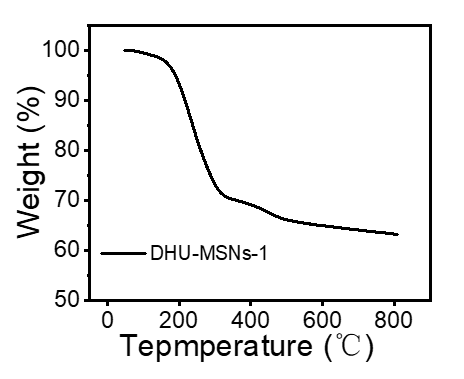


**Figure S8** Thermogravimetric curve of DHU-MSNs-1.

**Figure S9** (a) Fluorescence spectra of DHU-MSNs-1 (20 µg mL^−1^) before/after the addition of different concentrations of HOCl (0, 1, 2, 5 and 10 µM). (b) Time-dependent fluorescence intensity changes of DHU-MSNs-1 (20 µg mL^−1^) at 686 nm upon addition of 10 μM HOCl. (c) Fluorescence intensity of DHU-MSNs-1 (20 µg mL^−1^) at 686 nm after treatment with HOCl (10 μM) and amino acids (400 µM) (from B to K: Cys, Ala, His, Thr, Glu, Leu, Lys, Arg, Asp, Gly). (d) Fluorescence intensity of DHU-MSNs-1 (20 µg mL^−1^) at 686 nm after treatment with HOCl (10 μM) and different ions (400 µM) (from B' to I': NH_4_^+^, K^+^, SO_4_^2−^, F^-^, Mg^2+^, Na^+^, NO_2_^−^, Ca^2+^). (e) Fluorescence intensity of DHU-MSNs-1 (20 µg mL^−1^) at 686 nm after treatment with HOCl (10 μM) and ROS/RNS (20 µM) (from B''to I'': H_2_O_2_, ·OH, TBHP, ROO·, NO, O_2_^·−^, t-BuOO·, ONOO^−^). (f) LCMS plot of DHU-MSNs-1 (20 µg mL^−1^) after response with 10 µM HOCl.


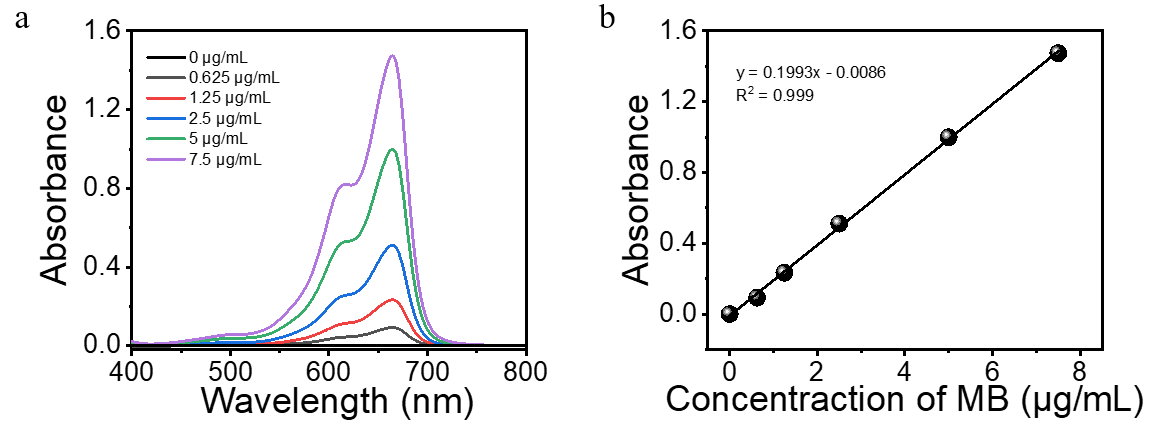


**Figure S10** (a) UV-vis absorption of different concentration of MB in PBS (7.4), (b) MB standard curve with absorption measured at 668 nm.


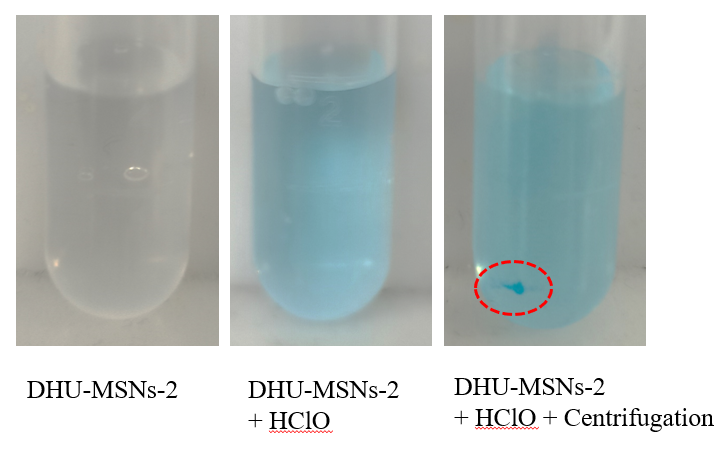


**Figure S11** Photograph of MB release.

**Figure. S12** (a) TEM image, (b) BET nitrogen adsorption/desorption isotherms (inset: BJH pore size distributions), (c) wide-angle XRD spectra of DHU-MSNs-2, (d) EDS analysis (scanned elements are C, N, O, Si, S) of DHU-MSNs-2, (e) DLS plot of DHU-MSNs-2.


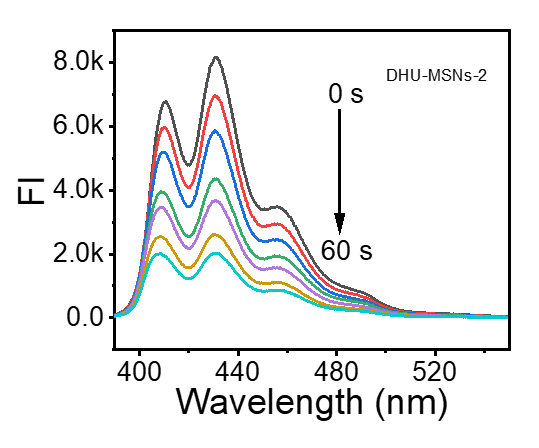


**Figure S13** Time-dependent fluorescence spectra of ABDA upon irradiation with 658 nm laser (0.3 W cm^−2^) in the response of DHU-MSNs-2 (100 µg mL^−1^) and HOCl.

**Figure S14** (a) Fluorescence spectra of DHU-MSNs-2 (20 µg mL^−1^) after response to different concentrations of HOCl (0, 1, 2, 5 and 10 μM). (b) Fluorescence intensity of DHU-MSNs-2 (20 µg mL^−1^) at 686 nm after treatment with HOCl (10 μM) and amino acids (400 µM) (from B to Q: Phe, Trp, cys, Ala, His, Val, Tyr, Thr, Glu, Ser, Leu, pro, Lys, Arg, Asp, Gly). (c) Fluorescence intensity of DHU-MSNs-2 (20 µg mL^−1^) at 686 nm after treatment with HOCl (10 μM) and different ions (400 µM) (from B' to K': CH_3_COO^−^, NH_4_^+^, K^+^, SO_4_^2-^, F^−^, Mg^2+^, NO_2_^−^, ClO_4_^−^, CO_3_^2−^, Ca^2+^). (d) Absorption spectra of DHU-MSNs-2 (20 µg mL^−1^) before and after response with 10 µM HOCl. (e) LCMS plot of DHU-MSNs-2 (20 µg mL^−1^) after response with 10 µM HOCl. Cell viability of (f) L929 cells and (g) HGE cells after 12 h and 24 h incubation with different concentrations of DHU-MSNs-2.

**Figure S15** Live/dead fluorescence images of Staphylococcus aureus after receiving various treatments: (a) PBS group; (b) DHU-MSNs-2 group; (c) DHU-MSNs-2+HOCl group; (d) DHU-MSNs-2+HOCl+Laser group.


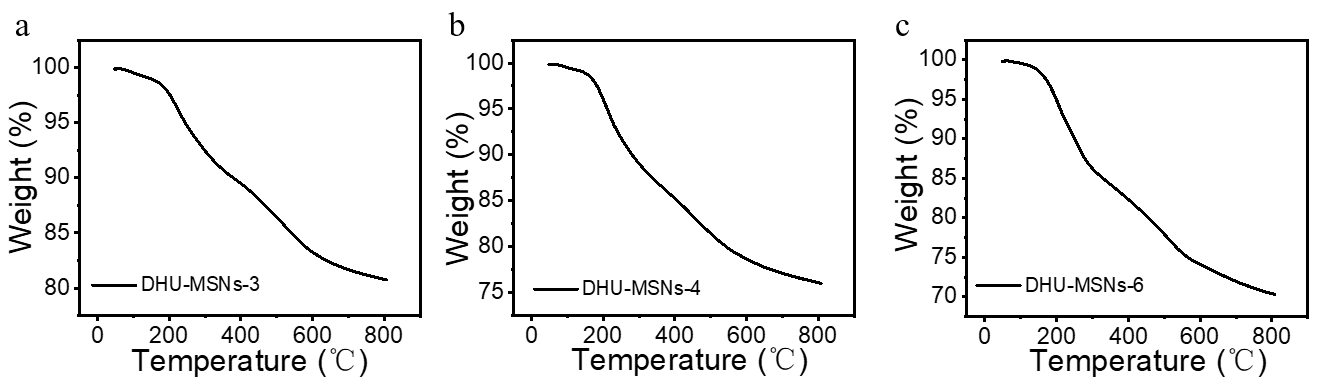


**Figure S16** Thermogravimetric curve of (a) DHU-MSNs-3, (b) DHU-MSNs-4, and (c) DHU-MSNs-6.


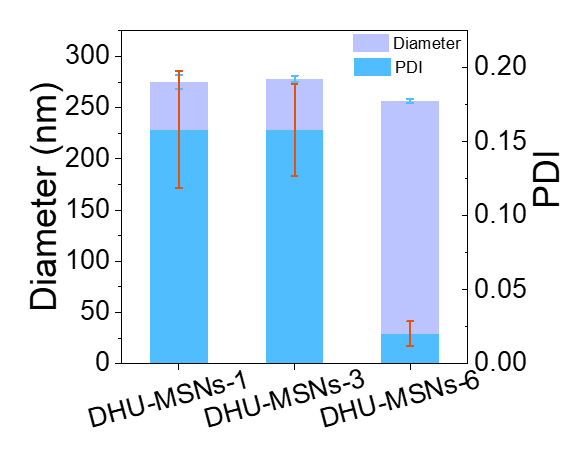


**Figure S17** Hydrated particle size and PDI of DHU-MSNs-1, DHU-MSNs-3, and DHU-MSNs-6.


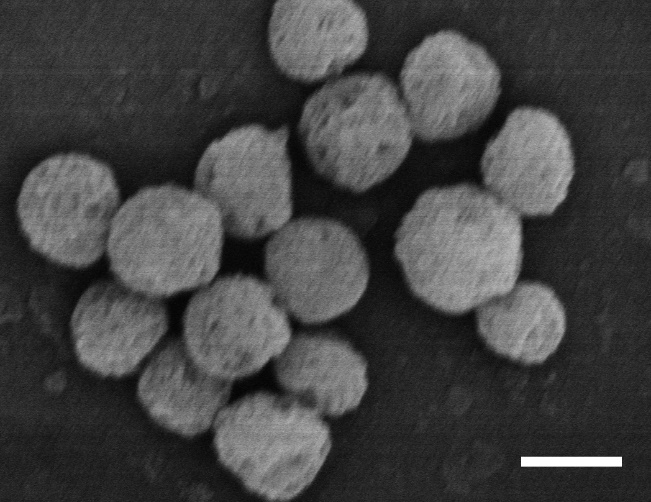


**Figure S18** SEM image of DHU-MSNs-6 (scale bar: 200 nm).

**Figure S19** (a) UV-vis absorption spectra of DOX, DHU-MSNs-1 and DHU-MSNs-6 in water. (b) DOX standard curve with absorption measured at 480 nm.

**Figure S20** (a) Fluorescence spectra of DHU-MSNs-6 (20 µg mL^−1^) before/after the addition of different concentrations of HOCl (0, 1, 2, 4, 6, 8 and10 µM). (b) Time-dependent fluorescence intensity changes of DHU-MSNs-6 (20 µg mL^−1^) at 686 nm upon addition of 10 μM HOCl. (c) Fluorescence intensity of DHU-MSNs-6 (20 µg mL^−1^) at 686 nm after treatment with HOCl (10 μM) and amino acids (400 µM) (from B to P: Phe, Trp, Ala, His, Val, Tyr, Thr, Glu, Ser, Leu, pro, Lys, Arg, Asp, Gly). (d) Fluorescence intensity of DHU-MSNs-6 (20 µg mL^−1^) at 686 nm after treatment with HOCl (10 μM) and different ions (400 µM) (from B' to N': CH_3_COO^−^, NH_4_^+^, K^+^, SO_4_^2−^, S_2_O_3_^2−^, F^−^, Mg^2+^, NO_2_^−^, ClO_4_^−^, CO_3_^2−^, HCO_3_^−^, Cu^2+^, Ca^2+^). (e) Fluorescence intensity of DHU-MSNs-6 (20 µg mL^−1^) at 686 nm after treatment with HOCl (10 μM) and other ROS (20 µM) (from B''to I'': H_2_O_2_, ·OH, TBHP, ROO·, NO, O_2_^·−^, t-BuOO·, ONOO^−^).


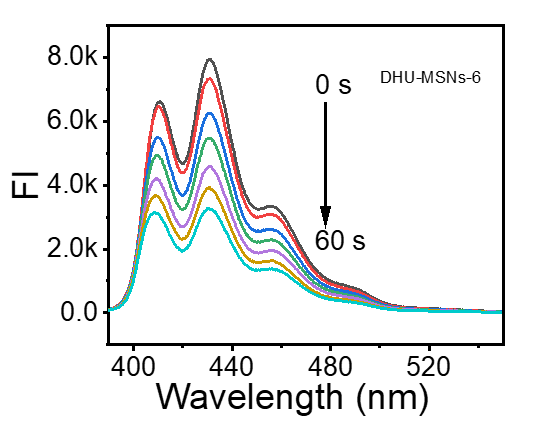


**Figure S21** Time-dependent fluorescence spectra of ABDA upon irradiation with 658 nm laser (0.3 W cm^−2^) in the response of DHU-MSNs-6 (100 µg mL^−1^) and HOCl.

**Figure S22** (a) In vitro single-linear oxygen generation capacity of DHU-MSNs-6 as a function of time. (b) Physical diagram of the color change of the solution before and after the reaction of DHU-MSNs-6 with HOCl. (c) LCMS plot of DHU-MSNs-6 (20 µg mL^−1^) after response with 10 µM HOCl.

**Figure S23** (a) photograph of red blood cells incubated with DHU-MSNs-6 with different concentrations after centrifugation. (b) UV-vis absorption spectra of DHU-MSNs-6 with different concentrations treated red blood cells.


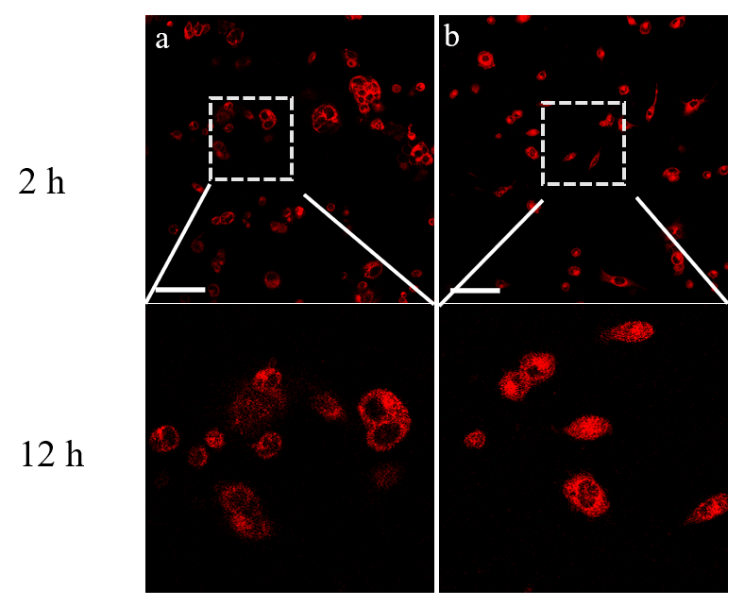


**Figure S24** Confocal laser scanning images of DHU-MSNs-6 (50 µg mL^−1^) co-incubated with A549 cells for (a) 2 h, (b) 12 h (scale bar: 50 μm).

**Figure S25** Flow cytometry analysis of the response of nanocomposites in A549 cells. (a) Flow cytogram. (b) Quantitative plots of flow cytometry results. Group 1-4 are DHU-MSNs-4, DHU-MSNs-4+HOCl, DHU-MSNs-6, DHU-MSNs-6+HOCl, respectively.


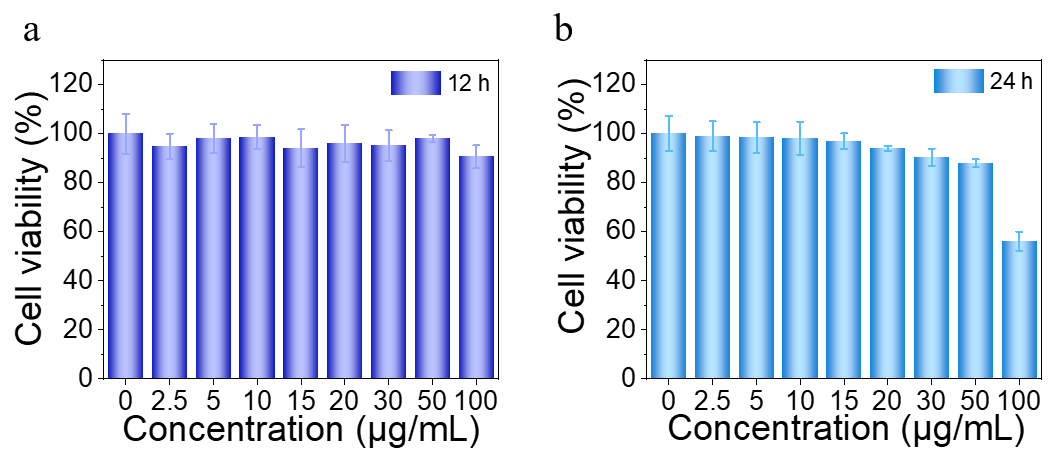


**Figure S26** Cell viability of A549 cells with different concentrations of DHU-MSNs-4 after incubation of (a)12 h and (b)24 h.


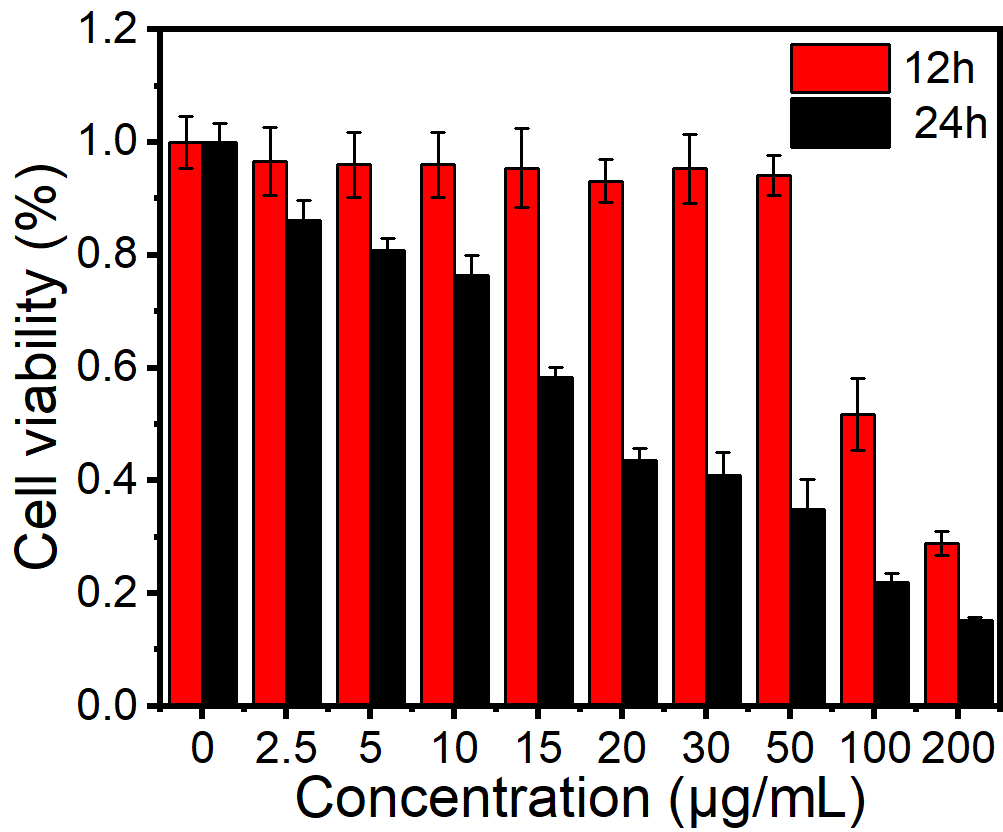


**Figure S27** Cell viability of A549 cells after 12 h and 24 h incubation with different concentrations of DHU-MSNs-6.


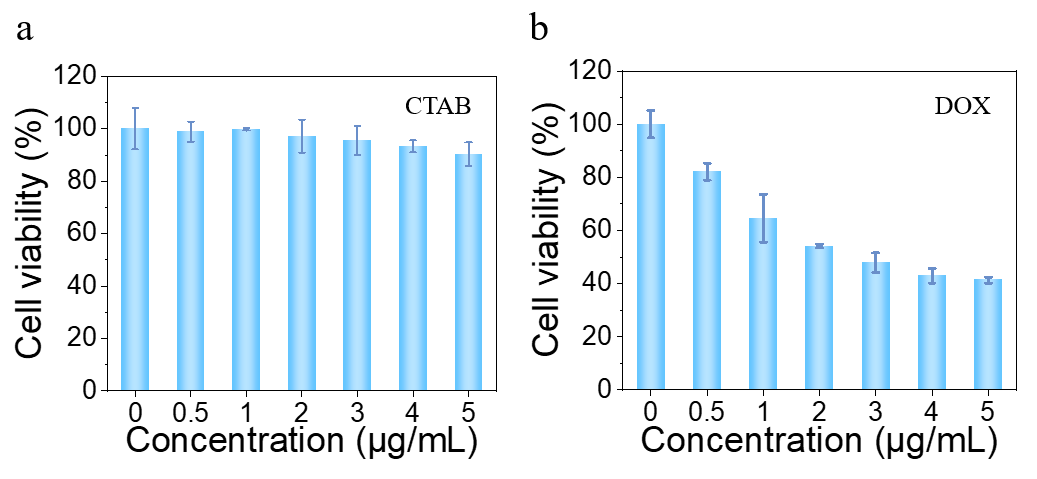


**Figure S28** Cell viability of A549 cells after 24 h incubation with different concentrations of (a) CTAB and (b) DOX.

**Figure S29** Live/dead fluorescence images of A549 cells after receiving various treatments. Group 1: Control; Group 2: DHU-MSNs-4; Group 3: DHU-MSNs-4+Laser 658 nm; Group 4: DHU-MSNs-6; Group 5: DHU-MSNs-6+Laser 658 nm.


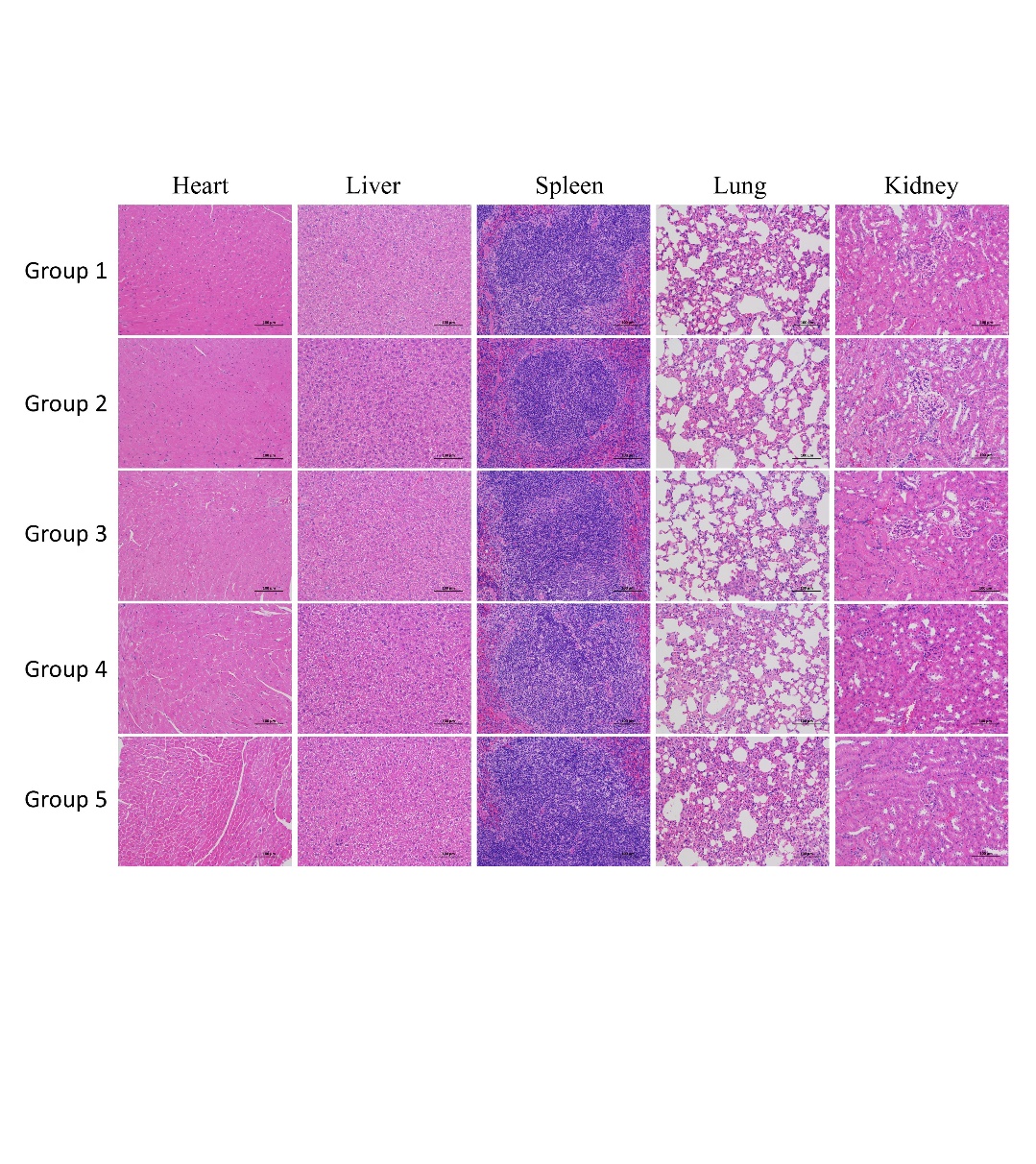


**Figure 30** H&E staining images of major organs of mice after 14 days of treatment.
